# Redox Modulation and Diffusion Kinetics in Ni-Doped CuO: Insights from Quantum-Inspired Electrochemical Methods

**DOI:** 10.1021/acsami.5c26218

**Published:** 2026-04-21

**Authors:** Tsung-Te Lin, Shih-Lung Yu, Yi-En Wu, Hai Yen Thi Nguyen, Wei-Lun Li, Yun-Syuan Liang, Kai-Hsun Lin, Ming-Kang Ho, Tsu-En Hsu, Krishtappa Manjunatha, Yi-Ru Hsu, Wei-Che Lo, Chia-Liang Cheng, B. Daruka Prasad, B. K. Monika, Hanumanthappa Nagabhushana, Meng-Chu Chen, Sheng Yun Wu

**Affiliations:** † Department of Mechanical and Systems Engineering, 63131 National Atomic Research Institute, Taoyuan 325207, Taiwan; ‡ Department of Physics, National Dong Hwa University, Hualien 97401, Taiwan; § Arete Honors Program, 34914 National Yang Ming Chiao Tung University, Hsinchu 300093, Taiwan; ∥ Department of Applied Science, 63285 National Taitung University, Taitung 950, Taiwan; ⊥ National Synchrotron Radiation Research Center, Hsinchu 30076, Taiwan; # Department of Physics, National Tsing Hua University, Hsinchu 30013, Taiwan; ∇ Department of Physics, BMS Institute of Technology and Management, VTU Belgavi Affiliated, Bangalore 560064, India; ○ Prof. C.N.R. Rao Centre for Advanced Materials, Tumkur University, Tumkur 572 103, India

**Keywords:** Ni-doped CuO nanoparticles, pseudocapacitors, electrochemical kinetics, R2WLD analysis, defect engineering

## Abstract

This study employs a comprehensive electrochemical methodology to evaluate the electrochemical kinetics of Ni-doped CuO nanoparticles synthesized via the solution combustion method. Working electrodes were prepared by coating a composite slurry onto nickel foam and tested in a three-electrode system with a 3 M KOH electrolyte. Cyclic voltammetry (CV), galvanostatic charge–discharge (GCD), and electrochemical impedance spectroscopy (EIS) were employed to investigate charge storage mechanisms and interfacial dynamics. A key advancement is the use of the R^2^-Window Linear Discharge (R2WLD) method to segment GCD curves and extract linear and pseudocapacitive discharge regions with high fidelity. Additionally, nonlinear Kernel Principal Component Analysis (KPCA) was employed to classify electrochemical regimes based on scan rate behavior. To interpret the evolution of discharge symmetry, a 1D Ising model was adapted to model energetic state transitions under increasing perturbation. These findings underscore the critical role of defect and lattice engineering in tuning the functional response of correlated oxide systems.

## Graphical abstract

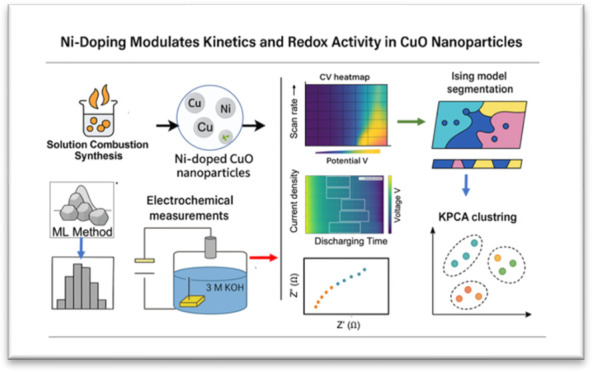

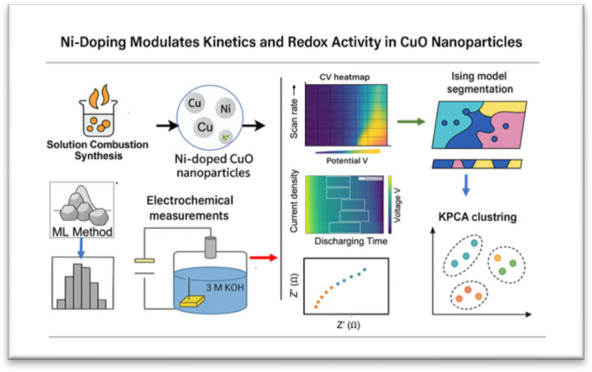

## 1 Introduction

Electrochemical energy storage has become a pivotal component in the global transition toward sustainable technologies. Supercapacitors, particularly pseudocapacitors, bridge the gap between batteries and traditional capacitors by delivering high power density, fast charge–discharge cycles, and long-term cycling stability.^1,2^ These attributes are essential for applications such as electric vehicles, wearable electronics, and grid-scale energy storage.

Transition metal oxides (TMOs) have emerged as leading candidates for pseudocapacitor electrodes due to their diverse oxidation states and favorable redox kinetics. Among them, copper oxide (CuO) stands out for its environmental friendliness, low cost, and relatively high theoretical specific capacitance (∼374 *F*·*g* ^–1^).^3^ With a narrow bandgap (∼1.2 eV) and abundance in the Earth’s crust, CuO offers an attractive platform for charge storage devices.^4^ Despite these merits, pristine CuO suffers from drawbacks including poor electrical conductivity, limited ion transport, and suboptimal cycling durability. To address these limitations, researchers have pursued strategies such as nanostructuring, hybridization with carbonaceous materials, and, most notably, cation doping.^5^

Aliovalent doping in CuO, especially with divalent cations such as Ni^2+^, has demonstrated significant enhancement in pseudocapacitive behavior. Ni^2+^ doping introduces strain due to its smaller ionic radius (0.69 *Å*) compared to Cu^2+^ (0.73 *Å*), resulting in lattice distortions that can enhance electron hopping pathways.^6^ Additionally, Ni contributes active redox pairs (Ni^2+^/Ni^3+^), which increases the density of faradaic reactions and improves charge storage capability.^7^ Furthermore, Ni doping enhances the stability of CuO structures during electrochemical cycling, limiting structural degradation and enhancing long-term performance.^8^ These effects make Ni-doped CuO a promising material for next-generation energy storage devices.

Several challenges remain in optimizing doped CuO materials. First, excessive dopant concentrations can lead to the formation of secondary phases or defect clustering, thereby reducing electrochemical performance.^9^ Second, morphology control during synthesis is crucial to prevent agglomeration and maximize the accessible surface area. A significant research gap lies in understanding how varying Ni concentrations influence the complex interplay between structure, conductivity, and pseudocapacitive kinetics. While cyclic voltammetry (CV) and galvanostatic charge–discharge (GCD) provide macroscopic insights, they fall short in distinguishing surface-limited from diffusion-limited contributions.^10^ Electrochemical impedance spectroscopy (EIS) offers a more detailed picture, but interpreting its parameters, such as charge transfer resistance and Warburg diffusion, requires careful modeling. Advanced tools such as equivalent circuit fitting and constant phase element (CPE) analysis are increasingly used to reveal internal resistive behaviors.^11^ Moreover, a standardized framework for quantifying and comparing pseudocapacitive materials is lacking. Many studies report capacitance values without accounting for electrode geometry, electrolyte diffusion rates, or current collector effects, leading to discrepancies across the literature.^12^

In recent years, solution combustion synthesis (SCS) has gained popularity for producing doped metal oxide nanoparticles. It allows rapid, scalable synthesis with good control over composition and crystallinity. Several studies have successfully synthesized Ni-doped CuO using SCS, showing enhanced electrochemical performance relative to pristine CuO.^13^ Analytical advances have paralleled these synthetic improvements. The *R* ^2^-Window Linear Discharge (R2WLD) technique has emerged as a powerful method for segmenting GCD curves into kinetic zones, offering new insights into discharge symmetry and reversibility. Likewise, machine learning methods, such as unsupervised Kernel Principal Component Analysis (KPCA), have been used to map the relationship between doping levels, morphology, and kinetic regimes. New physical models have also been introduced to describe capacitive behavior. For example, 1D Ising models, traditionally used in statistical physics, have been adapted to model transitions between capacitive and diffusive domains under changing scan rates or doping levels.^14^ These combined advances in synthesis, characterization, and modeling are ushering in a more rigorous, predictive approach to supercapacitor design.

Despite these developments, comprehensive studies that integrate structural, morphological, and electrochemical analyses of Ni-doped CuO remain limited. Few investigations have systematically explored the effects of varying Ni concentrations (e.g., 0–5%) on lattice contraction, defect generation, or electrochemical symmetry. This study addresses that gap by synthesizing a series of Ni-doped CuO nanoparticles using a controlled SCS process. The materials were analyzed using synchrotron-based XRD with Rietveld refinement, FE-SEM with machine learning-assisted particle size distribution analysis, Raman spectroscopy, and XPS. Electrochemical evaluation included CV, GCD with R2WLD segmentation, and EIS. In addition, nonlinear KPCA and a 1D Ising framework were applied to classify the kinetic regimes emerging from different dopant concentrations. These tools enable quantitative mapping of performance transitions from bulk-diffusion-dominated to surface-controlled behavior. The motivation for this work is two fold: to offer a reproducible methodology for understanding dopant effects in pseudocapacitive oxides, and to identify an optimal Ni concentration that maximizes electrochemical efficiency without compromising structural integrity.

## 2 Materials and Methods

### 2.1 Synthesis Method

Nickel-doped copper oxide (Cu_1–*x*_Ni_*x*_O) nanoparticles were synthesized using a solution combustion synthesis (SCS) technique, chosen for its efficiency in producing homogeneous, nanostructured metal oxides. Analytical-grade copper nitrate trihydrate [Cu­(NO_3_)_2_·3H_2_O] and nickel nitrate hexahydrate [Ni­(NO_3_)_2_·6H_2_O] were employed as metal precursors, while urea (CO­(NH_2_)_2_) served as the fuel and complexing agent. The redox stoichiometry was balanced according to propellant chemistry guidelines to ensure a self-sustaining combustion process.

For a typical synthesis, stoichiometric amounts of Cu­(NO_3_)_2_·3H_2_O and Ni­(NO_3_)_2_·6H_2_O were weighed based on the desired Ni doping level (x) and dissolved in deionized water along with a calculated amount of urea. The mixture was stirred thoroughly at room temperature to yield a clear, homogeneous solution. The solution was then gradually heated to ∼ 90*°C* to promote water evaporation, leading to the formation of a viscous gel as solvent volume reduced. The resulting gel was transferred to a preheated muffle furnace maintained at 500*°C* under ambient air conditions. Upon insertion, the gel underwent a rapid and exothermic combustion reaction within minutes, generating voluminous gases (primarily CO_2_, N_2_, and H_2_O) and producing a lightweight, foamy ash composed of Ni-doped CuO. This self-propagating reaction eliminated the need for prolonged heating at this stage, ensuring uniform dopant dispersion at the atomic level.

The as-synthesized powder was gently ground to reduce soft agglomerates and then calcined at 650*°C* for 3 h in air to improve crystallinity, enhance phase purity, and eliminate residual organic species. After cooling to room temperature, the product was reground into a fine powder. The final Ni-doped CuO nanopowders exhibited uniform morphology, phase stability, and high surface area, making them ideal candidates for subsequent structural, spectroscopic, and electrochemical characterizations relevant to supercapacitor applications.

### 2.2 Sample Characterizations

A suite of advanced characterization techniques was employed to assess the structural, morphological, vibrational, and electronic properties of the synthesized CuO and Ni-doped CuO nanoparticles. X-ray diffraction (XRD) measurements were conducted using a STOE StadiVari diffractometer at the National Synchrotron Radiation Research Center (NSRRC), Hsinchu, Taiwan. The high-resolution synchrotron source enabled the precise determination of lattice parameters, crystallite size, and phase composition. Rietveld refinement was applied to extract structural information, providing insight into doping-induced lattice distortions and phase evolution.

Field-emission scanning electron microscopy (FE-SEM) was performed using a JEOL JSM-7000F field-emission scanning electron microscope at National Dong Hwa University. SEM imaging allowed for the visualization of particle morphology, surface texture, and agglomeration behavior. A machine learning–aided segmentation approach was employed to obtain statistically robust particle size distributions. Energy-dispersive X-ray spectroscopy (EDX), integrated with SEM, confirmed the elemental composition and successful incorporation of Ni into the CuO lattice, with no detectable impurities.

Raman spectroscopy was performed using a WITec alpha300R confocal Raman microscope equipped with multiple laser sources. A 488 nm laser was used in this study to excite vibrational modes while minimizing fluorescence. Spectra were acquired at room temperature under ambient conditions, covering the range of 100–1500 cm^–1^. Peak shifts, broadening, and intensity changes with increasing Ni content were used to evaluate lattice strain and defect generation, in alignment with group theory and DFT predictions. X-ray photoelectron spectroscopy (XPS) was employed to investigate the chemical states of Cu, Ni, and O. High-resolution spectra revealed the oxidation states of Cu^2+^ and Ni^2+^ through characteristic multiplet splitting and satellite features. The binding energy shifts further indicated electronic structure modifications due to Ni incorporation.

Together, these techniques offered a multidimensional understanding of the structural and electronic modifications induced by Ni doping in CuO. The resulting data serve as a critical foundation for interpreting electrochemical behavior and performance trends in the subsequent analyses.

### 2.3 Supercapacitor Characterization

The working electrodes were fabricated by homogeneously blending Ni-doped CuO nanoparticles, carbon black, and polyvinylidene fluoride (PVDF) in a weight ratio of 80:10:10, using *N*-methyl-2-pyrrolidone (NMP) as the solvent. The resulting slurry was cast onto precleaned nickel foam substrates (1 cm × 1 cm), followed by drying at 80*°C* for 12 h. To minimize interfacial resistance, the electrodes were lightly pressed after drying, a protocol established for optimizing contact in oxide-based pseudocapacitors.

Electrochemical performance was evaluated in a standard three-electrode configuration, comprising an Ag/AgCl (saturated KCl) reference electrode, a platinum wire as the counter electrode, and a 3 M KOH aqueous solution as the electrolyte. The choice of KOH is motivated by its high ionic conductivity and wide electrochemical stability window in aqueous systems. Cyclic voltammetry (CV) was performed using a Squidstat Plus potentiostat over a scan rate range of 5–50 *mV*·*s* ^–1^ within a fixed potential window. Galvanostatic charge–discharge (GCD) measurements were conducted at current densities ranging from 1 to 5 *A*·*g* ^–1^. Specific capacitance, energy density, and power density were derived from GCD data using established electrochemical equations. To isolate intrinsic electrode kinetics and enable composition-resolved comparisons, the present work focuses on three-electrode measurements; two-electrode (symmetric/asymmetric) device assembly and long-term cycling stability evaluation are identified as important future work.

Electrochemical impedance spectroscopy (EIS) was performed over a frequency range of 1 Hz to 1 MHz with a 5 mV amplitude perturbation at the open-circuit potential. Nyquist plots were modeled using a dispersion-aware equivalent circuit that included elements for series resistance (*R*_*s*_), charge-transfer resistance (*R*_*ct*_), and Warburg impedance. Constant phase elements (CPE) were employed to account for nonideal capacitive behavior arising from surface roughness and heterogeneity. The integration of CV, GCD, and EIS techniques provided a holistic understanding of the pseudocapacitive behavior, ion transport dynamics, and electrochemical stability of Ni-doped CuO systems. Collectively, the data demonstrate that nickel incorporation improves charge-transfer kinetics and enhances cycling durability, underscoring the material’s potential for high-efficiency supercapacitor applications.

## 3 Characterizations and Analysis

### 3.1 XRD Rietveld Refinement Analysis of Pure and Ni-Doped CuO Nanoparticles

The crystal structure has a significant impact on the electrochemical performance of CuO and Ni-doped CuO, as revealed by CV, GCD, and EIS measurements. Changes in lattice parameters, crystallite size, and defect concentration influence ion diffusion, charge transfer resistance, and pseudocapacitive behavior, thereby directly affecting specific capacitance, energy density, and overall electrode kinetics. The Rietveld refinements in Figure 1a (undoped CuO) and Figure 1b (5% Ni-doped CuO) provide a quantitative view of the structural integrity and phase evolution of the CuO lattice upon Ni incorporation. Using a pseudo-Voigt profile function within the FullProf-2k refinement suite,^15^ the experimental diffraction profiles (red traces) are reproduced by the calculated patterns (black circles) with excellent fidelity, as evidenced by the very low and featureless difference curves (blue lines). The refined goodness-of-fit indicators *R*_*wp*_, *R*_*p*_, and χ^2^, listed in Table S1 (Supporting Information), fall within the typical range reported for nanocrystalline transition-metal oxides,^16^ confirming that the structural models are well constrained and essentially free of systematic mismatch between observed and calculated intensities. In both cases, the dominant phase is monoclinic tenorite CuO (space group *C*2/*c*), while the Ni-doped sample exhibits only weak additional reflections that can be indexed to a minor NiO secondary phase, indicating that a substantial fraction of Ni is accommodated substitutionally, while a minor fraction segregates as NiO within the CuO matrix. The accompanying crystal-structure renderings in Figure 1c and 1d visualize these models: undoped CuO is depicted with Cu (orange) and O (red) sublattices arranged in slightly distorted square-planar CuO_4_ coordination units interconnected through a mixture of corner- and edge-linkages, whereas in the 5% Ni-doped CuO model a fraction of the Cu sites is replaced by Ni (green), illustrating how Ni enters the monoclinic tenorite framework while preserving the overall lattice topology.

**Figure 1:**
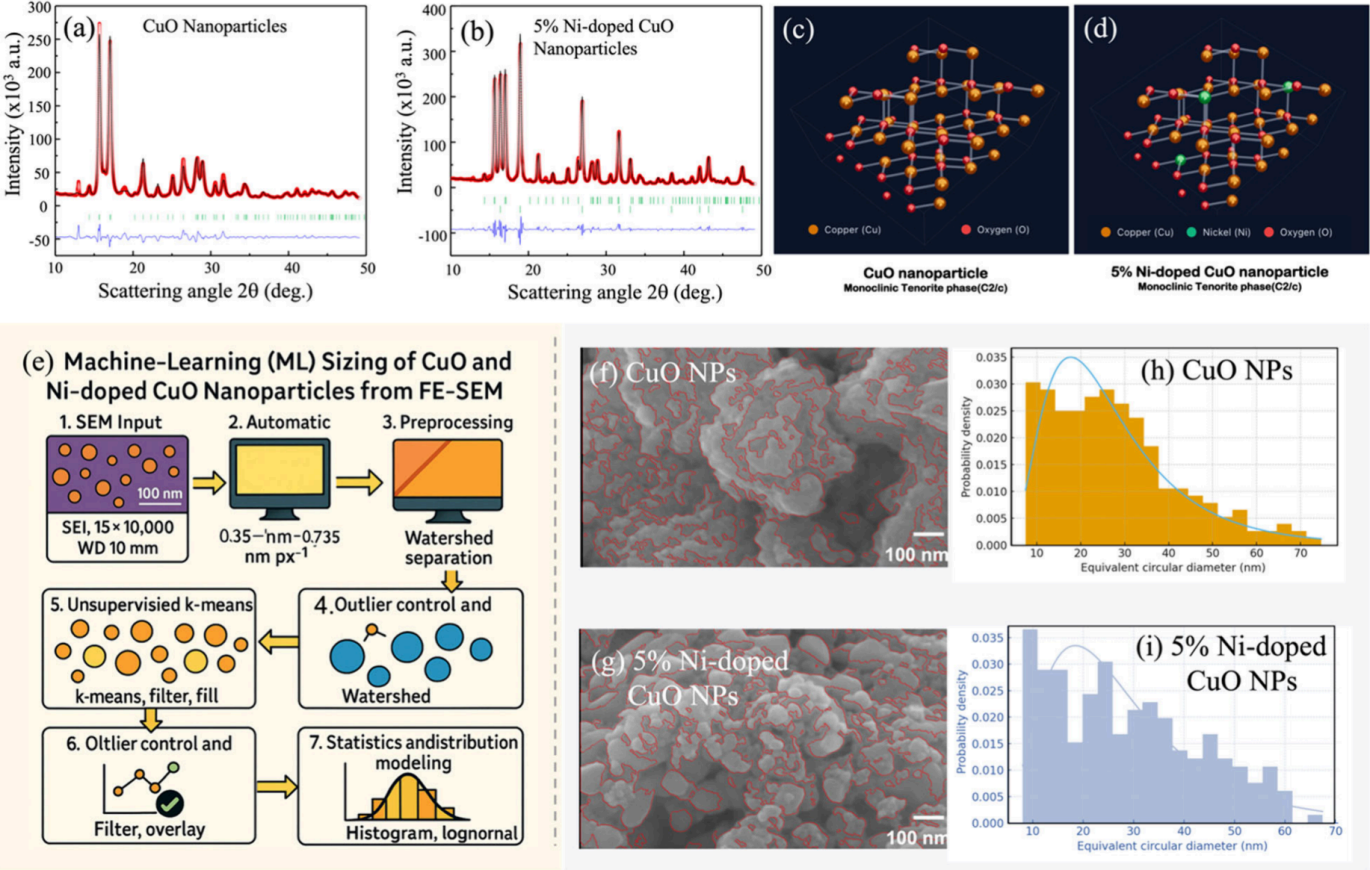
Rietveld-refined XRD patterns and structural/size analysis of CuO-based nanoparticles. (a) CuO and (b) 5% Ni–CuO: measured (red), fitted (black), Bragg positions (green), and difference (blue), confirming monoclinic tenorite CuO (*C*2/*c*) for both samples with a minor NiO secondary phase in the doped material. (c–d) Crystal-structure models showing Ni (green) substitution on Cu sites. (e) Machine-learning workflow for FE-SEM particle sizing (preprocessing → watershed separation → k-means segmentation → outlier/QC → distribution modeling). (f–g) SEM images with watershed boundaries (red). (h–i) Particle-size distributions with log-normal fits for CuO and 5% Ni–CuO.

The pristine CuO sample (Figure 1a) exhibits diffraction peaks exclusively indexed to the monoclinic tenorite phase (space group *C2/c*, JCPDS #48-1548), confirming its single-phase nature. The refined lattice parameters: *a* ≈ 4.6874(4) *Å*, *b* ≈ 3.4268(3) *Å*, *c* ≈ 5.1280(5) *Å*, and β ≈ 99.319(4)°, yield a unit cell volume of ∼81.289(12) *Å* ^3^, aligning closely with reported bulk values.^17^ The sharp, symmetric peaks and minimal microstrain values obtained from profile analysis indicate high crystallinity and a negligible defect density in the undoped system. In contrast, the 5% Ni-doped CuO sample (Figure 1b) requires a biphasic model for accurate refinement, incorporating both the CuO phase and a minor NiO secondary phase (space group *Fm3̅m*). The emergence of distinct reflections at 2θ ≈ 43.3° within the measured diffraction range (10–49.15°) is consistent with the onset of NiO crystallization, indicating partial phase segregation due to limited Ni solubility in the CuO host matrix. Quantitative phase analysis based on the Hill–Howard method^18^ indicates that the NiO secondary phase constitutes approximately ∼4 wt % (≤5 wt %) in the x = 5% Ni-doped sample, while the remaining majority phase is monoclinic CuO. Such a small fraction suggests that most Ni ions are incorporated substitutionally within the CuO lattice, with only limited phase segregation occurring near the solubility limit. From an electrochemical standpoint, this minor NiO fraction is expected to reside predominantly at grain boundaries or particle surfaces. Under alkaline conditions, NiO can undergo surface hydration to form the Ni­(OH)_2_/NiOOH redox couple, which may introduce additional interfacial pseudocapacitive contributions. However, due to its low abundance, this secondary phase does not dominate the composition-dependent electrochemical trends observed in the present study, which are primarily governed by Ni-induced lattice distortion, defect formation, and the resulting modulation of charge-transfer and diffusion kinetics in the CuO host matrix.

Doping-induced variations in the lattice parameters (*a* = 4.6890(2) *Å*, *b* = 3.4328(1) *Å*, *c* = 5.1378(2) *Å*, β = 99.342(3)°) reveal a slight increase in the unit-cell volume from 81.289(12) to 81.603(5) *Å* ^3^ upon 5% Ni substitution. Although Ni^2+^ (0.69 *Å*) is marginally smaller than Cu^2+^ (0.73 *Å*), the observed expansion indicates that local lattice relaxation, dopant-induced strain, and defect-mediated distortions outweigh the simple ionic-size effect.^19^ Such counterintuitive volume changes are well documented in transition-metal oxides, where substitutional doping can introduce octahedral tilting, bond-angle deviations, and weak anisotropic strain fields. The small increase in V therefore reflects enhanced structural disorder and local rearrangement of the Cu–O coordination environment rather than a size-driven contraction.

The Ni-doped sample additionally exhibits noticeable broadening of Bragg reflections, signifying increased microstrain and refinement of nanocrystalline domains.^20^ From a solid-state perspective, the incorporation of Ni^2+^ into the CuO lattice generates localized distortions and electronic potential inhomogeneities stemming from the ionic radius mismatch and altered bonding environment. These perturbations produce anisotropic strain fields manifested through peak broadening and subtle shifts of key reflections, particularly the (111) and (−111) planes, toward higher 2θ values. Although the monoclinic angle β increases only slightly (99.319(4)° → 99.342(3)°), the combined behavior of line broadening and peak shifting indicates the presence of localized compressive regions surrounding Ni-substituted sites. Such short-range structural distortions have significant implications for the material’s functional behavior, as they can modify Cu–O–Cu superexchange pathways and thereby influence charge-carrier mobility, redox kinetics, and spin-ordering phenomena.^21,22^

As shown in Table S1 (Supporting Information), the 5% Ni-doped CuO exhibits slightly elevated *R*_*wp*_ and *R*_*p*_ values due to the increased complexity of the two-phase model and peak asymmetry. Nevertheless, the overall χ^2^ values remain within acceptable limits, affirming the robustness of the refinement. The pseudo-Voigt function effectively captures both instrumental broadening and microstructural contributions, such as strain- and size-induced peak broadening, with residuals displaying no systematic deviation, indicating an accurate model.^23^ The Rietveld analysis reveals that low-level Ni doping in CuO retains the monoclinic tenorite framework while inducing subtle lattice expansion and structural disorder. The limited presence of NiO suggests the system approaches the solubility limit of Ni^2+^, delineating the transition from a solid-solution regime to a heterogeneous composite state. These structural effects, governed by ionic radius mismatch and defect chemistry, are likely to modulate the electronic structure, magnetic interactions, and catalytic behavior of the material, underlining the profound interdependence between atomic-scale structural evolution and macroscopic physical properties in transition metal oxides.^24,25^

### 3.2 Machine Learning Assisted SEM Nanoparticle Sizing

Figure 1e illustrates a transparent computer-vision workflow for extracting particle-size statistics from FE-SEM images of CuO and 5% Ni-doped CuO, acquired at 15 kV, × 100,000, WD = 10 mm. The pipeline is designed to be parameter-light, auditable, and replicable across data sets. First, the physical scale is calibrated automatically by detecting the 100 nm scale bar, yielding a pixel size of 0.735 nm px^–1^ for this image. Second, local contrast is normalized using CLAHE to suppress low-frequency background gradients while enhancing grain boundaries.^26^ Third, unsupervised k-means clustering in intensity space labels bright nanograins against the darker matrix; morphological opening/closing removes speckle and fills small holes to stabilize the segmentation.^27^ Fourth, a watershed transform applied to the Euclidean distance map conservatively splits contacting grains, thereby mitigating bias from agglomerates and sintering necks.^28^ Finally, connected components are measured to obtain the equivalent-circular diameter *d*_*eq*_ (ECD, $d_{eq}=2\sqrt{A/\pi }$ ), major/minor axes, and eccentricity; extreme outliers (>99th-percentile area) are discarded as likely multiparticle conglomerates. The resulting ECD distributions are modeled with a log-normal law, which is physically appropriate for multiplicative growth/fragmentation processes typical of oxide nucleation and coarsening.^29^ Group differences are assessed nonparametrically using the Mann–Whitney U test to avoid Gaussian assumptions.^30^

Figure 1f-g shows the undoped and 5% Ni-doped CuO nanoparticles’ surface. The micrograph is dominated by a granular matrix punctuated by broader terrace-like plates. The red overlay (watershed boundaries) tracks the intensity ridges that mark particle edges; note the continuity of boundaries across shallow gray level changes and the deliberate avoidance of oversegmentation on broad facets, indicating that our distance-map peaks are set conservatively. Pores and intra-agglomerate voids appear as darker channels, especially near the center, where connected watershed contours encircle lobed particles, a signature consistent with diffusion-limited growth and subsequent neck formation. Because segmentation is unsupervised, no hand-tuned thresholds are required; the only structural prior is that grains are locally brighter than the embedding shadow, a condition satisfied across both panels at 15 kV.

To model the size distribution, we adopt the standard log-normal form for particle diameters *d*:

1 $$f\left(d|\mu ,\sigma \right)=\frac{1}{d\sigma \sqrt{2\pi }}\;\exp\left[-\frac{{\left(\ln\;d-\mu \right)}^{2}}{2\sigma^{2}}\right]$$

where μ and σ are the mean and standard deviation of *lnd*. The geometric mean and multiplicative geometric standard deviation are *d*_*g*_ = exp (μ) and *GSD* = exp (σ), respectively. This parametrization directly connects to solid-state growth kinetics: σ summarizes the breadth of multiplicative noise along nucleation and coarsening pathways, while shifts in μ capture systematic changes in the typical grain size with processing or composition.^29^ All fitted parameters were summarized in Table S2 (Supporting Information).

Applying the pipeline to Figure 1f and 1h (CuO) yields 226 particles after QC. The central tendency is D50 = 25.41 nm, with D10 = 10.85 nm and D90 = 48.38 nm; the arithmetic mean is 27.76 nm with SD = 14.64 nm. The log-normal fit returns μ = 3.1801 and σ = 0.5522 for ln (*d*/1 *nm*), i.e., geometric mean 24.05 nm and geometric spread ×1.74. Kolmogorov–Smirnov (KS) goodness-of-fit indicates an adequate representation (D ≈ 0.055; p ≈ 0.49). The right-hand tail (≥50 nm) corresponds morphologically to the broad plates visible in Figure 1f; watershed segmentation limits their contribution by avoiding artificial splitting of planar terraces.

For the 5% Ni-doped CuO panel (Figure 1g and 1i), the same pipeline identifies 221 particles. We obtain D50 = 26.68 nm, D10 = 10.79 nm, D90 = 50.26 nm; the arithmetic mean is 28.88 nm with SD = 14.58 nm. The fitted log-normal parameters are μ = 3.2214 and σ = 0.5541 for ln (*d*/1 *nm*), corresponding to geometric mean 25.06 nm and geometric spread **×**1.74. The KS test again supports the model (D ≈ 0.062; p ≈ 0.35). Importantly, σ is essentially unchanged upon Ni substitution, implying that the multiplicative dispersion in growth/coarsening noise is comparable in both compositions.

To quantify the intergroup shift in the full distributions, we apply the Mann–Whitney U test (two-sided), obtaining U = 23674.5, p = 0.342, with rank-biserial effect size *r*_*rb*_ = 0.052. Thus, while the median increases by ∼1.3 nm upon Ni doping, the shift is not statistically significant and the effect size is small, indicating that the 5% Ni substitution does not substantially perturb the mesoscale pathways, nucleation rate, growth kinetics, or coarsening barrier that determine grain size under these synthesis conditions. Consequently, any composition-dependent changes in electrochemical or magnetic response are unlikely to be driven by gross particle-size differences; rather, they should originate in crystallographic/defect degrees of freedom (e.g., cation distribution and valence, octahedral distortions, strain fields, or surface states), which SEM-based sizing intentionally factors out.

Methodologically, the advantages are threefold. (i) Reproducibility: automatic scale detection and unsupervised segmentation remove operator bias and hidden thresholds. (ii) Physical interpretability: reporting both quantiles (D10/D50/D90) and log-normal (μ, σ) ties descriptive statistics to kinetic narratives of oxide growth.^29^ (iii) Quality control: conservative watershed markers and outlier filtering prevent overcounting on plates and under-counting in dense agglomerates, visible in the overlays of Figure 1f-g. This combination provides a robust bridge between imaging and property modeling, enabling fair comparisons across doped and undoped oxides.

### 3.3 Energy-Dispersive X-ray Spectroscopy (EDX) Analysis

Energy-dispersive X-ray spectroscopy (EDX) was carried out to verify the elemental composition and confirm the successful incorporation of Ni into the CuO lattice. Figure 2a-b presents representative EDX spectra of undoped CuO and 5% Ni-doped CuO nanoparticles, respectively. In the spectrum of the undoped sample (Figure 2a), distinct peaks appear at ∼0.93 keV and ∼8.05 keV, corresponding to the *CuL*_α_ and *Cu K*_*α*_ lines, respectively. A weaker *Cu K*_*β*_ peak is also visible near ∼8.90 keV. The presence of a peak at ∼0.52 keV is attributed to the *O K*_*α*_ transition, confirming the presence of oxygen in the sample. These peaks are characteristic of CuO and confirm the synthesis of pure phase of CuO nanoparticles, free from any detectable impurities.^31^ The Ni-doped CuO spectrum (Figure 2b) retains all the Cu and O features observed in the undoped sample but shows additional peaks at ∼0.85 keV and ∼7.47 keV, corresponding to the *NiL*_*α*_ and *Ni K*_*α*_ transitions, respectively, along with a weaker *Ni K*_*β*_ line at ∼8.26 keV. These features confirm the successful doping of Ni into the CuO matrix.^32^ Importantly, no peaks associated with extraneous elements (e.g., Cl, C, or S from residual precursors) are detected, indicating the chemical purity of the samples post-synthesis.

**Figure 2:**
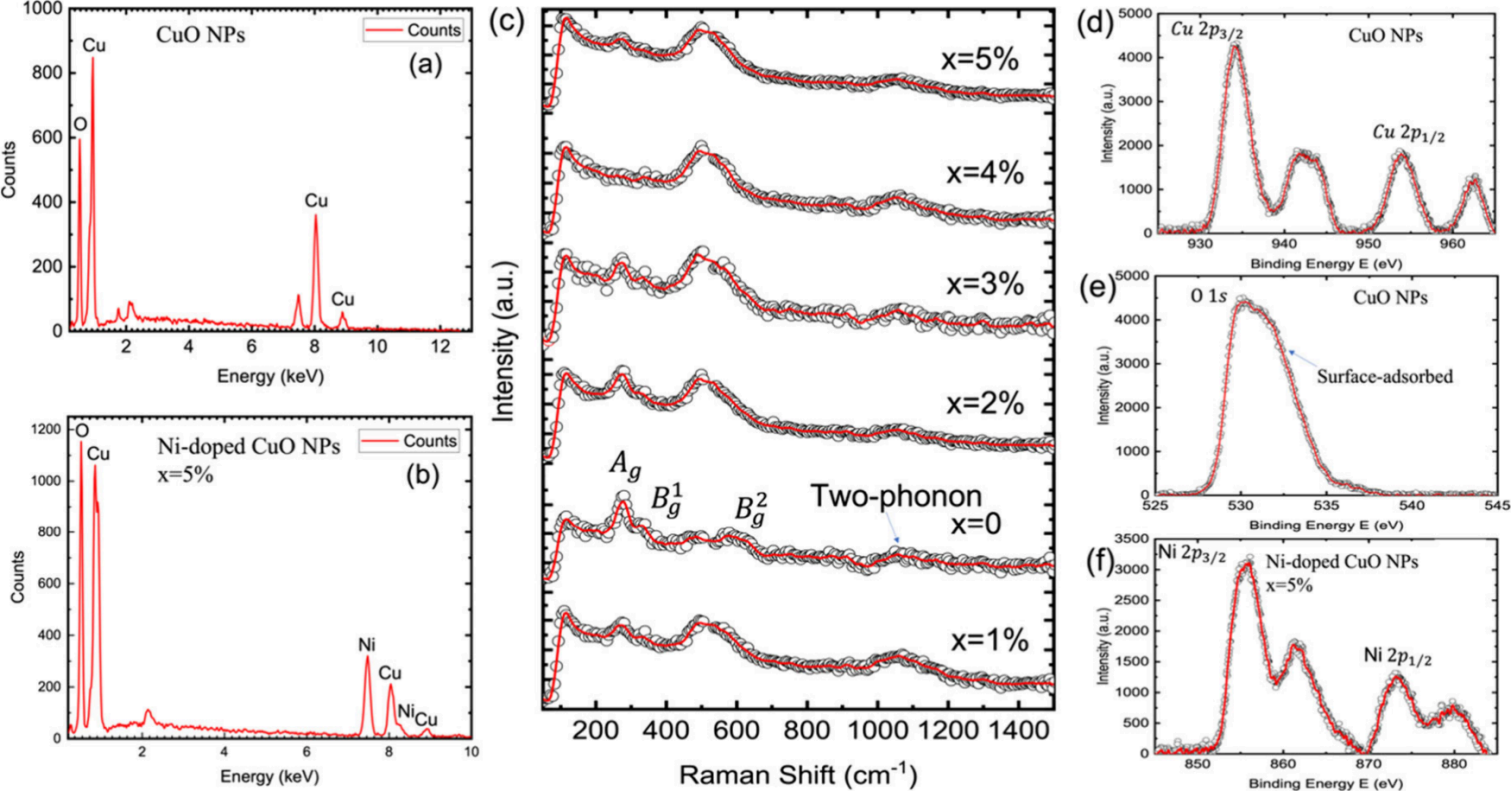
(a–b) EDX spectra of undoped and 5% Ni-doped CuO nanoparticles, showing only Cu and O for pristine CuO and the appearance of Ni peaks after doping, confirming impurity-free Ni incorporation. (c) Room-temperature Raman spectra (100–1200 cm^–1^) for x = 0–5% Ni-doped CuO: the monoclinic CuO modes at ∼297 (*A*_*g*_), 346 (*B*_*g*_^1^), and 629 cm^–1^ (*B*_*g*_^2^) progressively blue-shift and broaden with Ni content, and a weak two-phonon band near ∼1050 cm^–1^ develops, indicating substitutional doping and enhanced phonon–defect scattering without secondary phases. (d–f) High-resolution XPS core levels: Cu 2p with Cu^2+^ main peaks and shakeup satellites, O 1s dominated by lattice O^2–^ with a small surface/defect component, and Ni 2p consistent with Ni^2+^ in an octahedral environment. Black circles represent experimental data, and the red lines represent the fits.

While EDX is not inherently quantitative for light elements such as oxygen due to detector sensitivity and surface interaction limitations, the semiquantitative atomic percentages derived from the spectra indicate an increasing Ni content proportional to the nominal doping level. Table S3 (Supporting Information) summarizes the calculated atomic percentages of Cu, O, and Ni for undoped and Ni-doped samples. The data confirm that the Ni:Cu ratio increases as expected, verifying the systematic incorporation of x = 5% Ni-doping into the lattice, likely through the substitution of Cu^2+^ due to the similar valence and coordination environment.^19^

While EDX confirms the elemental presence of Ni, the high-resolution Rietveld refinement reveals that a minor NiO secondary phase (∼4 wt %) appears only at the highest doping level (x = 5%), indicating that the majority of Ni remains substitutionally incorporated in the CuO lattice. Previous reports have noted that when Ni exceeds the solubility limit or segregates, additional peaks near 37.2° (XRD) or ∼500 cm^–1^ (Raman) typically emerge;^33,34^ however, such features are absent in the present case, supporting homogeneous doping. The EDX results provide direct elemental confirmation of the chemical composition and validate the success of the Ni-doping strategy in CuO nanoparticles. These findings are consistent with structural and spectroscopic analyses, reinforcing the conclusion that Ni is incorporated into the CuO lattice in a substitutional manner.

### 3.4 Raman Spectroscopy Analysis

To probe lattice dynamics and assess symmetry perturbations in Ni-doped CuO nanoparticles, Raman spectroscopy was conducted across the doping range of 0–5%. The results are presented in Figure 2c and Table S4 (Supporting Information), covering the spectral range of 100–1200 cm^–1^. Interpretation of the vibrational features was supported by group theoretical analysis and compared with first-principles phonon calculations.

CuO crystallizes in a monoclinic structure with *C*_2*h*_^6^ (*C*2/*c*) symmetry and two formula units per primitive cell (*Z* = 2). According to group theory, the Γ-point vibrational modes of monoclinic CuO decompose as follows:^35^

2 $$\Gamma_{optical}=4A_{g}+5B_{g}+3A_{u}+4Bu$$

Of these, *A*_*g*_ and *B*_*g*_ modes are Raman-active, while *A*_*u*_ and *B*_*u*_ are infrared-active. Thus, 9 Raman-active modes are theoretically allowed, although typically only 3–5 strong modes are observed due to selection rules, instrumental resolution, and mode intensities. The three dominant modes in undoped CuO appear at 297 cm^–1^, 346 cm^–1^, and 629 cm^–1^, corresponding to the *A*_*g*_ (Cu–O bending), *B*_*g*_(1), and *B*_*g*_(2) (Cu–O stretching along [101]) phonon modes, respectively. These assignments are consistent with both experimental observations and density functional theory (DFT) phonon mode calculations,^34,36^ which place these modes at 295, 345, and 625 cm^–1^, respectively. With increasing Ni concentration, the following key trends are observed: (i) Blue shifts of Raman modes: All first-order phonon modes shift toward higher wavenumbers. Although the Rietveld refinement indicates a slight increase in the average unit-cell volume, the Raman blue shift reflects local bond stiffening around substitutional Ni sites,^19^ which may coexist with global lattice relaxation. The stretching mode at ∼629 cm^–1^, being most sensitive to bond length changes, exhibits a measurable shift of ∼6 cm^–1^ at 5% doping. (ii) Broadening of phonon lines: Peak broadening indicates increased phonon scattering, reflecting higher levels of structural disorder, point defects, and lattice strain. This is especially evident in the *B*_*g*_ modes, which are more sensitive to symmetry breaking. (iii) Reduced intensity and symmetry relaxation: The progressive reduction in Raman intensity and mode resolution reflects loss of long-range order and partial breakdown of Raman selection rules, which allows otherwise weak or symmetry-forbidden modes to appear weakly due to mode mixing or Brillouin zone folding.

A broad band centered near 1050 cm^–1^ is visible in all samples and grows with Ni content. As this mode lies outside the first-order phonon range of CuO, it is attributed to second-order (two-phonon) Raman scattering, possibly involving overtones or combinations of the *A*_*g*_ and *B*_*g*_ modes, such as

3 $$\omega_{1050}\approx \omega_{B_{g}\left(1\right)}+\omega_{B_{g}\left(2\right)},\;\mathrm{or}\;2\omega_{A_{g}}$$

This assignment is supported by previous theoretical and experimental reports of second-order features in CuO arising from phonon–phonon interactions.^34^ Alternatively, contributions from surface adsorbates (e.g., carbonate species) cannot be ruled out entirely; however, the absence of companion modes at ∼1410–1450 cm^–1^ reduces the likelihood of carbonate contamination.^32^

Recent DFT studies employing hybrid functionals or GGA+U approaches predict Raman-active modes for monoclinic CuO in excellent agreement with experimental values. For example, Bielecki et al.^34^ report *A*_*g*_ and *B*_*g*_ modes at 292, 341, and 624 cm^–1^, which closely match our experimental observations. The systematic shifts and disorder-induced features observed with Ni doping are consistent with predictions of phonon softening/hardening under local symmetry perturbation and chemical pressure.^36^ The integration of group theory and DFT phonon calculations provides a rigorous framework for interpreting the Raman spectral evolution of Ni-doped CuO. The observed blue shifts, mode broadening, and second-order scattering confirm that Ni substitution induces significant local distortion, enhances phonon–defect scattering, and modifies the vibrational density of states, all of which are crucial for understanding the resulting changes in thermal, electronic, and magnetic properties of the doped system.

### 3.5 X-ray Photoelectron Spectroscopy (XPS) Analysis

To investigate the elemental composition and electronic states within the Ni-doped CuO nanoparticles, X-ray Photoelectron Spectroscopy (XPS) was conducted. The high-resolution spectra for Cu 2p, O 1s, and Ni 2p core levels are presented in Figure 2d-**f**. These spectra provide direct information on the chemical state of each element, offering insight into the electronic structure modifications induced by Ni incorporation into the CuO lattice.

Figure 2d shows the Cu 2p spectrum, where two main peaks are observed at binding energies of approximately 932.5 eV (Cu 2*p*_3/2_) and 952.5 eV (Cu 2*p*_1/2_). These are consistent with the Cu^2+^ oxidation state in CuO.^37^ In addition to the main doublet, pronounced shakeup satellite features are observed around 940–945 eV and 960–965 eV. These shakeup satellites are characteristic of Cu^2+^ in a charge-transfer oxide and are indicative of significant hybridization between Cu 3d and O 2p orbitals.^38^ Such features are absent in Cu^+^ species, confirming the dominant Cu^2+^ state. The presence of these satellites supports the classification of CuO as a charge-transfer insulator, consistent with the Zaanen–Sawatzky–Allen (ZSA) framework.^22^ The spectral line shape and fitting accuracy also suggest a well-ordered local environment, with minimal inhomogeneity or metallic Cu contamination.

The O 1s core-level spectrum (Figure 2e) reveals a prominent peak centered at ∼530.2 eV, which corresponds to lattice oxygen (O^2–^) in the CuO matrix.^39^ This binding energy is attributed to oxygen atoms bonded within a transition metal oxide framework, where the O 2p states hybridize strongly with metal 3d orbitals.^40^ A weak shoulder at higher binding energy (∼531.5–532.5 eV) is observed and can be attributed to surface-adsorbed species such as hydroxyl groups, adsorbed water, or oxygen vacancies. These defects are often associated with local lattice distortions, which can give rise to midgap states and impact the material’s transport and optical properties.

The Ni 2p spectrum (Figure 2f) shows distinct peaks at ∼855 eV (Ni 2*p*_3/2_) and ∼873 eV (Ni 2*p*_1/2_), along with intense satellite features between 860–870 eV and 875–880 eV. This spectral profile is indicative of Ni^2+^ in an oxide environment, such as NiO.^41^ The presence of these multiplet structures and satellites confirms that Ni is not metallic but exists in a divalent state, likely within an octahedral ligand field. The successful incorporation of Ni^2+^ into the CuO lattice may result in local lattice distortions due to the ionic radius mismatch between Ni^2+^ and Cu^2+^, leading to a modified electronic structure and enhanced electron correlation effects. These alterations can significantly influence the material’s band structure, particularly through the introduction of localized states and modification of the Mott-Hubbard or charge-transfer character of the host CuO.^22,42^

The XPS analysis confirms the presence of Cu^2+^, Ni^2+^, and lattice O^2–^ in the Ni-doped CuO system. The clear observation of multiplet splitting and satellite structures is consistent with a strongly correlated oxide system. The incorporation of Ni into the CuO lattice modifies the local electronic environment, potentially leading to significant changes in charge transport, optical absorption, and magnetic properties, making the system promising for applications in catalysis, sensors, and spintronic devices.

## 4 Results and Discussion

### 4.1 Modulation of Electrochemical Kinetics in Ni-Doped CuO Nanoparticles

Cupric oxide (CuO), a p-type narrow-bandgap semiconductor, has garnered attention for its multifunctional properties in photovoltaics, catalysis, and particularly in energy storage systems, such as supercapacitors and lithium-ion batteries.^2,43^ Its redox activity and stability make it suitable for pseudocapacitive behavior; however, the relatively low conductivity limits its rate capability.^44^ Doping is a conventional strategy to tailor the electronic and defect properties of semiconducting oxides. In this context, Ni-doping is anticipated to modulate both the local electronic structure and ionic diffusion properties. Ni substitution introduces extrinsic dopant levels, modifies carrier concentration, and distorts the CuO lattice, phenomena that intricately affect charge transport, redox kinetics, and capacitive behavior. The aim of this study is to unravel how varying Ni concentration affects these properties using a combination of classical CV analysis and advanced modeling techniques.

#### 4.1.1 CV Profiles and Doping-Induced Modulation of Electrochemical Behavior

To clarify the multiple anodic and cathodic features observed in the Ni-doped electrodes, it is important to consider the coexistence of Cu- and Ni-centered redox processes in alkaline electrolyte. CuO-based pseudocapacitance generally proceeds through sequential Cu surface reactions involving Cu^2+^/Cu^+^ conversion and Cu^2+^/Cu^3+^-like oxyhydroxide formation. With Ni incorporation, an additional Ni^2+^/Ni^3+^ redox couple becomes electrochemically active following surface hydration. Accordingly, the lower-potential redox pair can be assigned primarily to CuO/Cu_2_O (or Cu–OH related) interconversion, while the higher-potential pair corresponds to CuOOH formation that partially overlaps with the Ni­(OH)_2_/NiOOH transition introduced by Ni doping. The apparent peak splitting and broadening therefore arise from the coexistence of Cu- and Ni-centered redox couples together with heterogeneous local electrochemical environments (surface, near-surface, and defect-rich regions), which distribute the redox potentials over a finite range rather than producing a single sharp Faradaic peak. A detailed assignment of the possible Cu- and Ni-centered redox reactions responsible for these features is provided in Note S1 (Supporting Information).

Figure 3a-f presents CV curves for CuO nanoparticles with 0 to 5% Ni-doping at scan rates from 5 to 50 *mV*·*s* ^–1^. Pristine CuO (x = 0%) shows distinct redox peaks attributable to Cu^2+^/Cu^+^ transitions, consistent with prior literature on Cu-based pseudocapacitors. As Ni content increases, peaks shift, broaden, and exhibit reduced symmetry, particularly at 4–5% doping. From the standpoint of electronic structure, Ni^2+^ (3d^8^) substitution for Cu^2+^ (3d^9^) leads to alterations in crystal field stabilization, band bending, and Fermi level shifting. These changes reduce the density of available Cu redox sites, explaining the peak broadening and intensity loss at higher doping levels. At moderate doping levels (2–3%), the redox peaks broaden slightly yet remain prominent, suggesting enhanced hybridization between Ni and Cu orbitals, which potentially leads to a more delocalized electron cloud, thereby benefiting redox reversibility. Furthermore, increasing the scan rate reveals the kinetic limitations of electron transfer, with the current response deviating from linearity in highly doped samples, indicative of resistive and diffusive bottlenecks induced by excessive lattice strain and charge carrier trapping.

**Figure 3:**
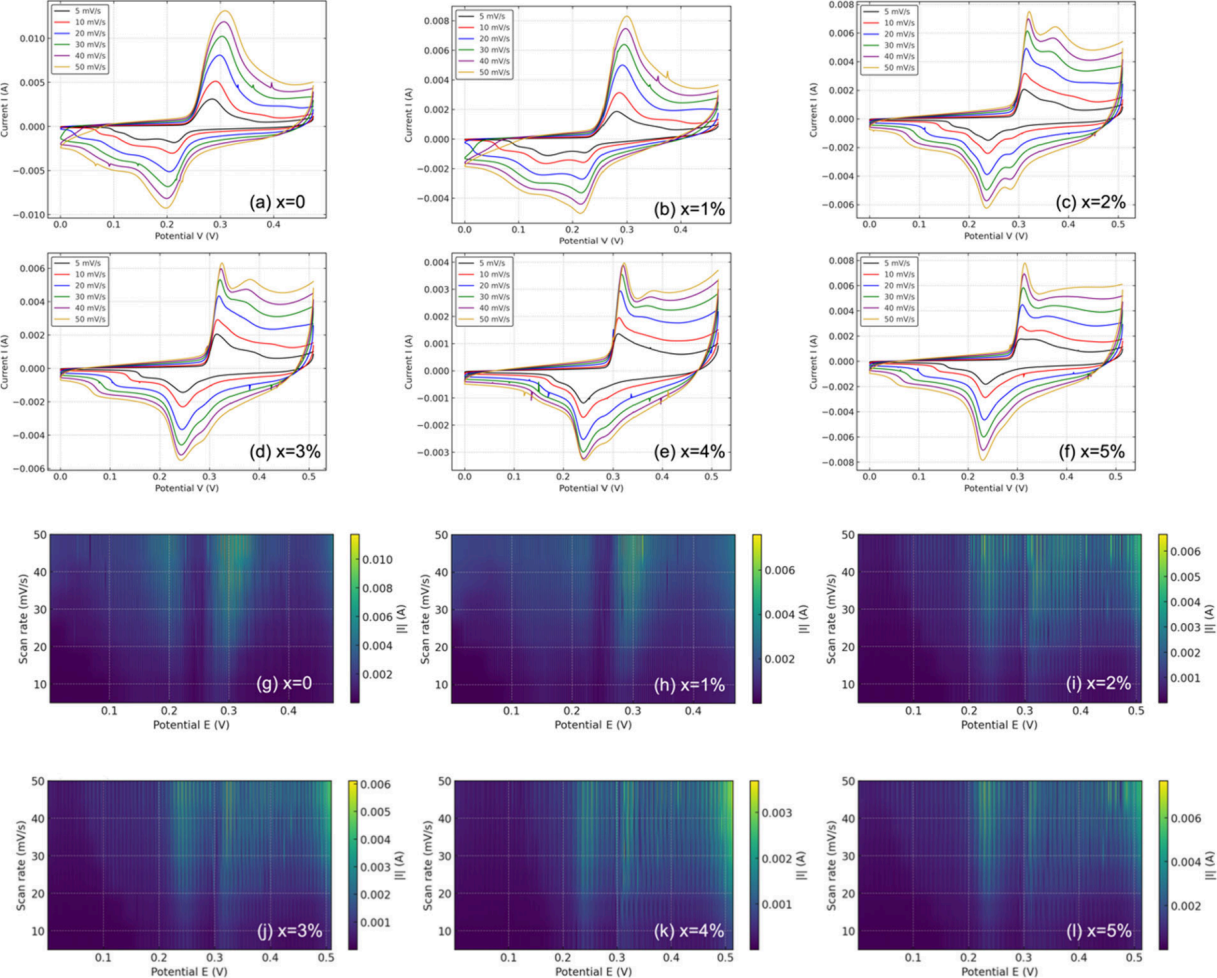
(a)–(f) CV curves of Ni-doped CuO (x = 0–5%) measured at scan rates of 5–50 *mV*·*s* ^–1^ within 0.0–0.55 V, showing doping- and rate-dependent redox responses. (g)–(l) Heatmaps of |*I*(*E*,ν)|versus potential and scan rate (5–50 *mV*·*s* ^–1^) for x = 0–5%, visualizing how Ni content reshapes the magnitude and distribution of electrochemical activity across the potential window.

#### 4.1.2 Electrochemical Activity Mapping via Current Heatmaps

Figure 3g–l visualizes the absolute current response |*I*(*E*,ν)| as a function of potential and scan rate. For x = 0, the current is concentrated around 0.3 V. As Ni doping increases, the heatmaps evolve to reveal broader current bands, particularly between 0.2–0.5 V for x = 2–3%. These observations indicate an increase in redox-active sites and a broadened energy distribution of surface states resulting from the incorporation of Ni. The smearing of current intensity with increased Ni content reflects a higher density of defect states and shallow traps that facilitate carrier hopping or polaronic transport, phenomena well-explained by Mott’s variable-range hopping model in disordered systems.^45^ This result supports the hypothesis that moderate Ni doping enhances the density of accessible states within the electrochemical window, improving the kinetics of ion-electron transfer reactions.

#### 4.1.3 Randles–Ševčík Analysis of Diffusion Behavior

To quantify the kinetic mechanism, we plotted the anodic peak current (*I*_*peak*_) versus the square root of the scan rate ( $\sqrt{\nu }$ ), shown in Figure S1a–f (Supporting Information). The strong linear relationships (*R* ^2^ > 0.99) across all doping levels validate the dominance of diffusion-controlled processes in the redox behavior of CuO. This diffusion dependence does not contradict pseudocapacitive behavior, because the CuO/NiOOH redox process still involves ion transport within near-surface regions, which can produce Randles–Ševčík-type scaling. However, the slope decreases progressively with increasing Ni content, indicating a reduction in effective diffusion coefficient (D) or electroactive surface area. The decreased slope in higher-doped samples is likely attributed to dopant-induced lattice disorder, which can impede long-range ionic diffusion due to enhanced carrier scattering or formation of insulating NiO domains at grain boundaries.^46^ Thus, while doping introduces favorable electronic changes, excessive substitution compromises long-range diffusion paths, highlighting the necessity for an optimal dopant threshold.

#### 4.1.4 Surface-Controlled Kinetics: Capacitive Contribution

Figure S2 (Supporting Information) illustrates the capacitive coefficient *k*_1_ across the potential range, extracted via the relationship *I* = *k*_1_ *v* + *k*_2_√*v*. The capacitive component, arising from surface-controlled fast redox reactions, peaks prominently in x = 2% and x = 3% samples. This enhancement is linked to changes in the surface electronic structure resulting from Ni substitution. Moderate Ni doping can improve surface state density and reduce charge transfer resistance (*R*_*ct*_), increasing double-layer capacitance and surface pseudocapacitance.^47^ Moreover, Ni-doped sites may act as catalytic centers, enhancing the reversibility of redox processes via improved electron affinity and orbital overlap with oxygen p-states. The data indicate that an optimal Ni content (∼2–3%) enhances capacitive contributions, rendering these compositions suitable for high-rate applications, such as supercapacitors.

#### 4.1.5 Diffusion-Controlled Component and Redox Peak Evolution

Complementary to the capacitive contribution, Figure S3 (Supporting Information) maps the diffusion-controlled coefficient *k*_2_, responsible for bulk-limited processes. At x = 0, we observe modest diffusion peaks around 0.3 V, while x = 2–4% doping shows sharp and intense peaks, particularly between 0.25–0.45 V. These features suggest Ni incorporation lowers the activation energy for ion intercalation, possibly via induced oxygen vacancies or lattice strain that open interstitial pathways. Ni dopants may also participate directly in redox reactions (Ni^2+^/Ni^3+^), contributing to total charge storage and explaining the additional peak intensity.^48^ However, at x = 5%, a notable decline in diffusion contribution is observed, indicating that overdoping leads to phase segregation or passivation effects that reduce bulk charge transport.

#### 4.1.6 Ising Model-Based Kinetic Regime Mapping and Quantum KPCA of Kinetic Variability

To obtain a physically coherent mapping of kinetic regimes along the electrochemical potential axis, the potential window is discretized into bins *E*_*i*_ and each bin is assigned a binary state *s*_*i*_ (e.g., *s*_*i*_ = +1 for capacitive-dominated behavior and *s*_*i*_ = −1 for diffusion-dominated behavior). The optimal segmentation is obtained by minimizing a one-dimensional Ising energy functional $H\left(s\right)=-J\underset{i}{\sum }s_{i}s_{i+1}-\underset{i}{\sum }h_{i}s_{i}$ , where *h*_*i*_ is a local field constructed from the continuous kinetic indicator extracted from CV analysis (such as the potential-dependent *b*-value or capacitive fraction), and *J* > 0 represents an effective nearest-neighbor coupling.

In this statistical-physics formulation, *J* acts as an interaction energy that penalizes rapid state transitions between adjacent potential bins, thereby enforcing spatial coherence of the kinetic regime along the potential axis. The parameter therefore controls the effective correlation length of the segmented regime map and suppresses spurious fluctuations arising from noise in the pointwise kinetic indicators. Importantly, this interaction energy does not correspond to a microscopic adsorption or Coulomb interaction between electrolyte ions. Instead, it is an effective regularization parameter, typically expressed in dimensionless form (or normalized by *k*_B_ *T*), used to extract physically meaningful domains from noisy electrochemical data. In this work, *h*_*i*_ is constructed directly from experimentally derived kinetic descriptors, such as potential-resolved *b*-values or capacitive fractions, allowing the Ising framework to convert continuous electrochemical indicators into a statistically robust domain segmentation of capacitive- and diffusion-controlled regimes.

To spatially resolve the nature of electrochemical kinetics, we employed an Ising spin model to segment potential regions into capacitive (spin = 1) and diffusive (spin = 0) states. Figure 4a–f shows that as the Ni content increases from 0% to 3%, the capacitive zones expand across the voltage window, particularly from 0.2 to 0.4 V. Beyond x = 4%, diffusion reemerges as the dominant mechanism. From a theoretical standpoint, this mapping mirrors phase transitions, with doping acting as an external field that shifts the system from an ordered (diffusion-dominated) to a disordered (capacitive-dominated) state. This is akin to a kinetic analog of ferromagnetic ordering, where capacitive alignment reflects cooperative surface activity. This modeling highlights the phase-like nature of charge storage behavior and provides a concise way to visualize regime transitions induced by chemical perturbation.

**Figure 4:**
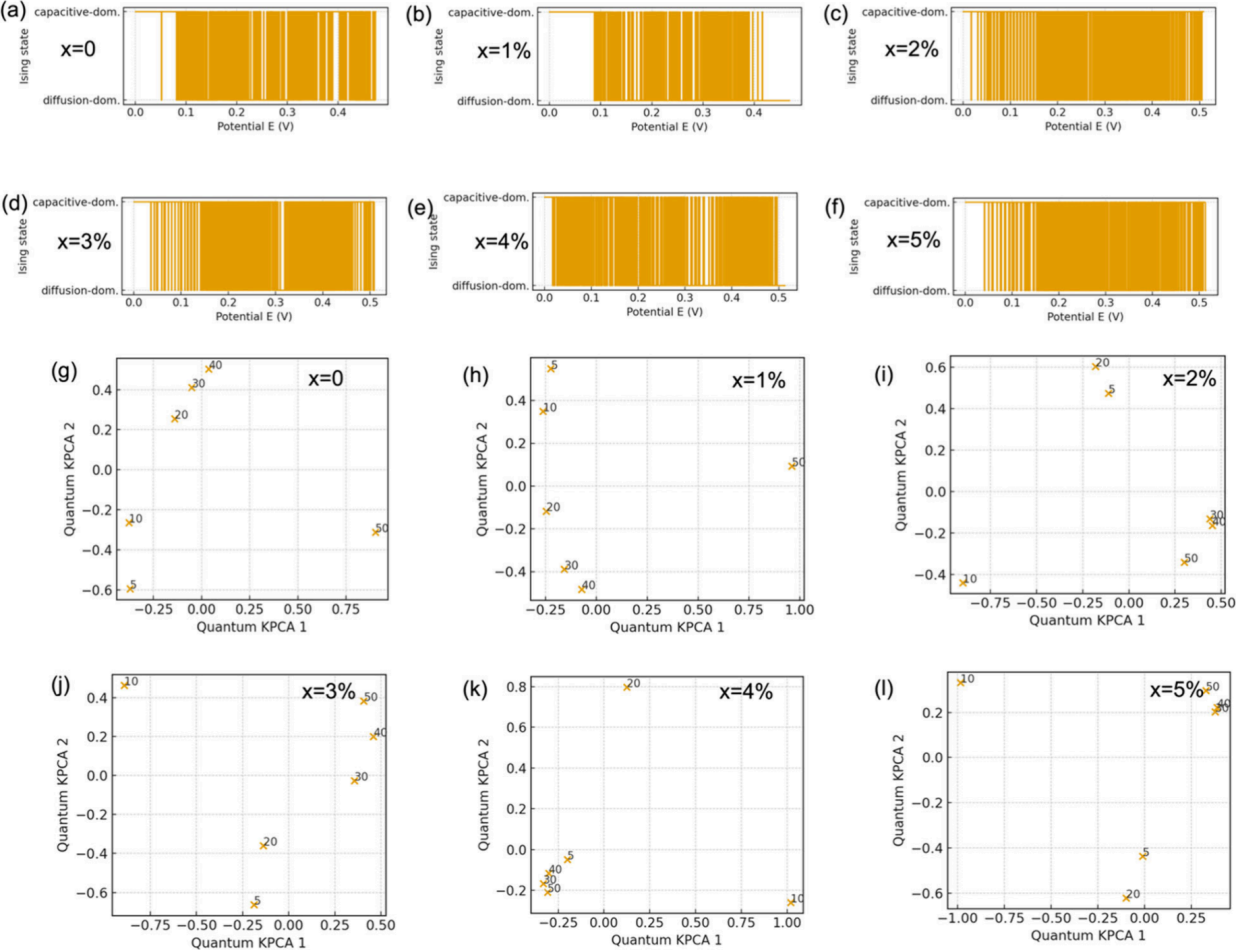
(a–f) Ising-model kinetic segmentation of Ni-doped CuO (x = 0–5%), mapping potential-dependent regions into capacitive-dominated (yellow) and diffusion-dominated (white) regimes and showing an expanded capacitive corridor at intermediate Ni contents (notably ∼0.2–0.4 V). (g–l) Quantum KPCA projections of CV-derived kinetic descriptors at scan rates 5–50 *mV*·*s* ^–1^ (labels), where doping-dependent clustering/separation captures the evolution of rate response and highlights the strongest kinetic distinction at intermediate Ni doping.

Figure 4g–l presents quantum kernel principal component analysis (KPCA) of CV-derived kinetic data, revealing nonlinear relationships not captured by traditional PCA. At x = 2%, scan rate points spread widely in the 2D projection space, suggesting maximal kinetic heterogeneity and optimal balance between capacitive and diffusive behavior. This analysis is similar in spirit to Brillouin zone folding in solid-state physics, where doping modifies symmetry and electronic band dispersion. Here, Ni acts as a symmetry-breaking agent, creating new local kinetic states that are interpreted as nonlinear manifolds in feature space. At higher doping (x = 5%), the system exhibits clustering reminiscent of kinetic degeneracy, implying reduced reactivity diversity, likely due to phase separation or carrier localization effects.

#### 4.1.7 Integrated Kinetic Summary and Optimal Doping Window

A comprehensive overview of the kinetic and electrochemical aspects is summarized in Figure S4 (Supporting Information). Specific capacitance vs scan rate reveals that pristine CuO exhibits the highest specific capacitance across the entire scan-rate range, as shown in Figure S4a, while x = 2–3% maintains superior performance at high scan rates, signifying improved rate capability. Heatmaps of *b*-values from *I*∝ν^*b*^ (Figure S4b) show transitions from diffusion-dominated (*b* ≈ 0.5) to capacitive-dominated (*b* ≈ 1.0) regimes near x = 3%. The capacitive fraction heatmap (at 50 mV/s) peaks between 0.2–0.4 V and x = 2–3%, affirming the findings from Figures S2 and S3, as shown in Figure S4c. The Ising-based regime map (Figure S4d) illustrates the expansion and subsequent contraction of capacitive zones beyond x = 3%, indicating the boundary of beneficial doping. Together, these observations point to an optimal doping window of x = 2–3%, where the electrochemical behavior achieves a synergistic balance between redox activity, carrier mobility, and kinetic accessibility.

This work examines the intricate relationship between dopant concentration, solid-state properties, and electrochemical kinetics in Ni-doped CuO nanoparticles. From a physicist’s lens, the modulation of pseudocapacitive behavior is not merely a surface phenomenon, it emerges from deep electronic structure alterations, defect engineering, and carrier transport tuning. Moderate Ni doping (x = 2–3%) optimally perturbs the CuO lattice, enhancing capacitive behavior while preserving sufficient bulk diffusion. The integration of Ising modeling and quantum KPCA provides a robust framework for visualizing and interpreting these transitions, reflecting core principles in statistical physics and nonlinear systems theory. These insights lay the groundwork for rational design of high-performance doped oxides and expand our understanding of doping-induced kinetic phenomena in energy materials.

### 4.2 Galvanostatic Charge–Discharge (GCD) and *R* ^2^-Window Linear Discharge (R2WLD) Analysis

The accurate determination of specific capacitance and kinetic performance in pseudocapacitive materials depends on identifying the truly linear region of the discharge curve, thereby minimizing subjective selection bias and curvature distortion. For the present Ni-doped CuO nanoparticle series (x = 0–5%), we applied the deterministic *R* ^2^-Window Linear Discharge (R2WLD) algorithm to extract unbiased linear slope segments from each galvanostatic discharge trace. Specific capacitance is obtained from the discharge trace *V*(*t*) by selecting a subinterval [*t*_*start*_,*t*_*end*_] over which *V* decreases approximately linearly and fitting *V*(*t*_*end*_) ≈ *V*(*t*_*start*_) – α­(*t*_*end*_ – *t*_*start*_) by least-squares, so that α = |[*V*(*t*_*end*_) – *V*(*t*_*start*_)]/(*t*_*end*_ – *t*_*staart*_)| and *C*_*sp*_ = *J*/α with *J* = *I*/*m* (*A*·*g* ^–1^, *m* the active mass). Because window choice strongly affects α (onset IR-drop and tail curvature bias the slope), we replace ad hoc selection with an automated procedure, the R2WLD method, which identifies the contiguous discharge segment maximizing *R* ^*2*^ under fixed constraints and then computes *C*_*sp*_ = *J*/*|α|* on that segment. Unlike conventional midpoint selection or fixed-percentage trimming, R2WLD scans all contiguous subwindows within the discharge, computing the linear regression and maximizing *R* ^2^ under predefined constraints (minimum window length ≥ 30% of discharge duration). The selected window, therefore, excludes the onset IR-drop and diffusion-limited tail while preserving the midregion where capacitive behavior dominates. A detailed description of the voltage heatmap and the selected fitting window method is provided in Note S2 (Supporting Information).

To assess linearity and ensure consistency, discharge voltage traces were normalized (time axis = 0 → 1) and mapped as heatmaps. Meanwhile, differential slope maps *dV*/*dt* highlight constant-slope regions. Regions of nearly zero curvature correspond to ideal capacitive domains, whereas green-to-blue areas indicate diffusion-limited or nonideal kinetics. The window boundaries identified by the *R* ^2^ optimization confirm the reproducibility of this algorithmic approach, revealing doping-dependent changes in discharge linearity and electrochemical symmetry.^49–51^ Once slope parameters were determined, energy and power densities were calculated from the R2WLD tabulated window bounds (*t*_*start*_, *V*_*start*_), (*t*_*end*_, *V*_*end*_), we calculated the window energy density:

4 $$E_{sp}=J\times V_{avg}\times \Delta t\left(J\cdot g^{-1}\right)$$

with *V*_*avg*_ = (*V*_*start*_ + *V*_*end*_)/2 and Δ*t* = *t*_*end*_ – *t*_*start*_, and the average power density:

5 $$P_{sp}=\frac{E_{sp}}{\Delta t}\left(W\cdot g^{-1}\right)$$

We report *E*_*sp*_ in *Wh*·*kg* ^–1^ via *E*_*sp*_ × (1000/3600), and *P*_*sp*_ in *kW*·*kg* ^–1^ (numerically equal to *W*·*g* ^–1^). This window-based evaluation provides a transparent, auditable path to correlate specific capacitance with composition and rate, independent of total discharge time or parasitic contributions.^52^

#### 4.2.1 Discharge Morphology and Pseudocapacitive Symmetry

Across all compositions, the GCD curves exhibit quasi-triangular morphologies characteristic of dominant pseudocapacitive charge storage (Figure 5a–f). The discharge time decreases systematically with increasing current density, while the small composition-dependent IR drops confirm the low internal resistance of the electrodes. Pristine CuO (x = 0) exhibits the longest discharge duration at a given current density, indicating the highest specific capacitance under galvanostatic conditions, whereas moderate Ni substitution (x ≈ 2–3%) results in slightly reduced discharge time but improved curve symmetry and voltage retention, signifying improved rate capability and more stable electron–ion transport coupling. The normalized voltage maps (Figures 5g–l) and the corresponding *dV*/*dt* slope maps (Figure S5a–f, Supporting Information) provide a direct visual representation of how the discharge linearity evolves with Ni content. For the undoped sample (x = 0%), the *R* ^2^- selected windows (white boxes) lie in relatively late and moderately broad segments of the discharge trace, reflecting the need to avoid both the initial IR drop and the pronounced curvature that develops near the low-voltage tail. Upon introducing Ni at low–intermediate levels (x ≈ 1–3%), these windows systematically shift toward slightly earlier normalized times and become more compact, especially at higher current densities. In this composition range, the bright, nearly vertical band in the voltage heatmaps is well-aligned across all *J*, signaling a well-defined pseudolinear regime in which charge storage is dominated by fast Faradaic and double-layer processes with reduced diffusion polarization. At higher Ni loading (x = 4–5%), the optimal windows broaden again and move back toward later discharge times, consistent with a progressive loss of ideal linearity and the emergence of slower processes, plausibly associated with defect clustering, local compositional disorder, or inhomogeneous current pathways.^53,54^

**Figure 5:**
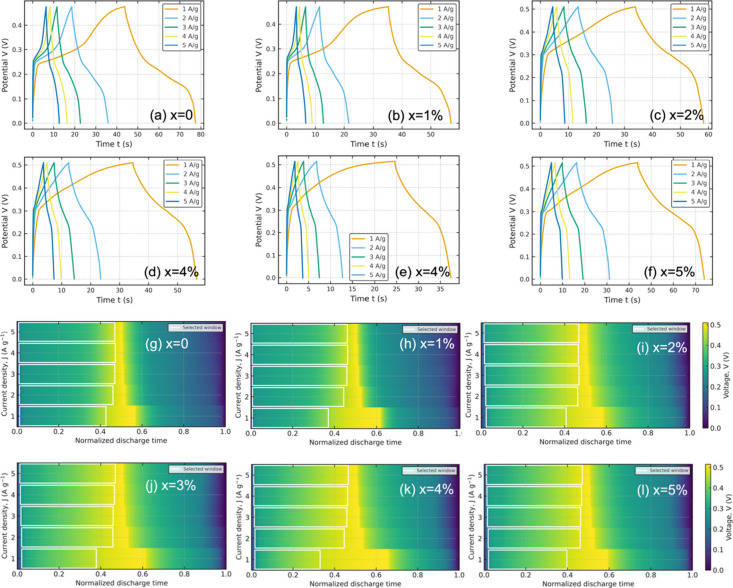
(a–f) GCD curves of Ni-doped CuO (x = 0–5%) at *J* = 1–5 *A*·*g* ^–1^, showing shorter discharge times at higher *J* and near-triangular profiles with small, composition-dependent IR drops, consistent with predominantly pseudocapacitive storage. Moderate Ni contents (∼2–3%) extend the discharge and reduce diffusion-related curvature compared with undoped CuO, indicating improved rate behavior. (g–l) Discharge heatmaps (voltage vs normalized time and *J*) for x = 0–5%; white boxes indicate the R^2^-optimized linear-discharge windows used to extract −d*V*/d*t* and *C*_*sp*_, positioned in mid-discharge to avoid the initial IR drop and the low-voltage tail, with window location/width varying systematically with *J* and Ni content.

The *dV*/*dt* maps reinforce this interpretation. For x = 2–3%, a wide midtime corridor of nearly uniform slope extends across the full current-density range, indicating that the effective capacitance is both time-independent and only weakly perturbed by kinetic limitations. In contrast, the x = 0 and 5% samples show strong variations of *dV*/*dt* at the beginning and end of discharge, which sharply restricts the time window over which a single linear slope can be defined. The nonmonotonic evolution of both the voltage and slope landscapes thus reveals an optimal Ni content around 2–3%, where electronic and ionic transport are jointly maximized, while under- and overdoping reintroduce nonlinear, diffusion-limited behavior.

#### 4.2.2 Quantitative R2WLD Capacitance Behavior

The R2WLD procedure identifies the most linear segment of each GCD discharge curve and converts the corresponding slope into a specific capacitance, providing an internally consistent evaluation of rate capability across all Ni compositions (Figures S6–S11 and Table S5, Supporting Information). The least-squares regression is therefore applied only to the *R* ^2^-optimized mid-discharge window (Figure S6; Note S2, Supporting Information). Any apparent mismatch between the fitted line and the full discharge curve arises from the intentionally excluded onset IR drop and diffusion-controlled low-voltage tail, and thus does not indicate poor fitting quality. Instead, these excluded regions correspond to polarization and ion-transport processes that would bias the capacitance estimation if included in the regression. This window-selection approach ensures that capacitance is extracted from the physically meaningful quasi-linear regime of the discharge profile while avoiding artifacts associated with polarization and diffusion-controlled curvature.

For undoped CuO (x = 0%), the fitted midtime windows produce specific capacitances of order ∼120 *F*·*g* ^–1^ at 1 *A*·*g* ^–1^, decreasing by only ∼10–15% at 5 *A*·*g* ^–1^. The relatively narrow windows in Figure S6 (Supporting Information) together with curvature at the beginning and end of discharge, indicate that a large fraction of the total potential drop is associated with interfacial polarization and slow ion rearrangement in the low-conductivity CuO matrix, consistent with earlier reports on binary transition-metal oxides.^55^ Introducing a small amount of Ni (x = 1%) lowers the absolute capacitance to values in the ∼80→55 *F*·*g* ^–1^ range as the current density increases from 1 to 5 *A*·*g* ^–1^ (Figure S7, Supporting Information), but the discharge becomes more nearly linear: the R2WLD windows are clearly defined, extend over a sizable fraction of the midtime region, and exhibit high *R* ^2^ at all *J*. This behavior indicates improved slope uniformity and suppressed diffusion curvature, consistent with more homogeneous electron–ion percolation facilitated by Ni-induced defect levels.^56^

At intermediate Ni levels (x = 2–3%), the system reaches an optimal compromise between capacitance magnitude and rate stability. For x = 2%, *C*_*sp*_ decreases only gradually from the upper-77 *F*·*g* ^–1^ at 1 *A*·*g* ^–1^ to the mid-65 *F*·*g* ^–1^ at 5 *A*·*g* ^–1^ (Figure S8, Supporting Information), whereas x = 3% shows a similarly modest drop from the upper-69 to the mid-56 *F*·*g* ^–1^ (Figure S9, Supporting Information). The associated R2WLD windows remain broad and centered in the mid-discharge region, signaling that the effective capacitance is nearly time-independent over the useful voltage range. These results, together with the voltage and slope heatmaps, indicate that moderate Ni incorporation optimizes rate stability and linear discharge behavior, placing x ≈ 2–3% in the “sweet spot” for pseudocapacitive CuO.^57^

Beyond this optimum, further Ni addition gradually degrades the rate response. At x = 4%, both the overall discharge time and the selected linear windows shrink (Figure S10, Supporting Information), and the specific capacitance drops to roughly 38 *F*·*g* ^–1^ at 1 *A*·*g* ^–1^ and ∼28 *F*·*g* ^–1^ at 5 *A*·*g* ^–1^. The contracted windows and stronger curvature are consistent with transport bottlenecks arising from dopant-induced lattice distortion, local strain fields, or partial segregation of NiO_x_-rich regions that obstruct continuous ion migration through the CuO host.^58^ In the most heavily doped sample (x = 5%), the absolute capacitance is significantly recovered: R2WLD analysis yields ∼97 *F*·*g* ^–1^ at 1 *A*·*g* ^–1^, decreasing to ∼77 *F*·*g* ^–1^ at 5 *A*·*g* ^–1^ (Figure S11, Supporting Information). The midtime windows remain well-defined across all currents, indicating that, despite increased structural complexity, Ni-rich domains provide additional electronically conductive pathways that compensate for some of the diffusion penalties. This nonmonotonic dependence of *C*_*sp*_ on Ni content reflects the competition between improved electronic conductivity and the onset of defect clustering or secondary-phase formation, phenomena widely documented in doped transition-metal oxides.^59^

#### 4.2.3 Composition-Dependent Rate Capability and Energy–Power Characteristics

We emphasize that these energy-density values are calculated for a single working electrode in a three-electrode cell over a narrow aqueous voltage window; thus, the absolute numbers are expected to be modest and should primarily be interpreted for relative comparison of doping-dependent kinetics (Table S6, Supporting Information). The composition-resolved rate capability curves extracted from the R2WLD fits (Figure 6a and Supporting Information of Table S6) show a clear hierarchy in *C*_*sp*_(*J*) across the Ni-doping series. Undoped CuO (x = 0) delivers the largest specific capacitance at all current densities, but its *C*_*sp*_ decreases noticeably as *J* increases, reflecting the stronger curvature and narrower linear windows seen in the voltage and slope maps. Introducing Ni progressively reshapes this behavior. The x = 1% electrode exhibits a substantially reduced capacitance and a relatively steep drop with *J*, indicating that, although the discharge becomes more linear, part of the redox-active CuO network is sacrificed. In contrast, the x = 2% and x = 3% samples display intermediate *C*_*sp*_ values with only moderate, nearly linear decreases from 1 to 5 *A*·*g* ^–1^, consistent with the broad midtime linear windows and nearly uniform d*V*/d*t* regions in Figures S6–9 (Supporting Information). These compositions thus combine reasonably high capacitance with robust rate stability. At a higher Ni content, x = 4%, the performance is the poorest: *C*_*sp*_ is the lowest of the series and decays rapidly with *J*, in line with the contracted fitting windows and stronger early/late-time curvature. For x = 5%, the capacitance partially recovers and remains the second highest over the measured current range, although still below that of undoped CuO. The persistent midtime linear segments and moderate *J*-dependence of *C*_*sp*_ in Figures S10–S11 (Supporting Information) suggest that Ni-rich pathways restore electronic connectivity without fully overcoming diffusion limitations at the highest doping level.

**Figure 6:**
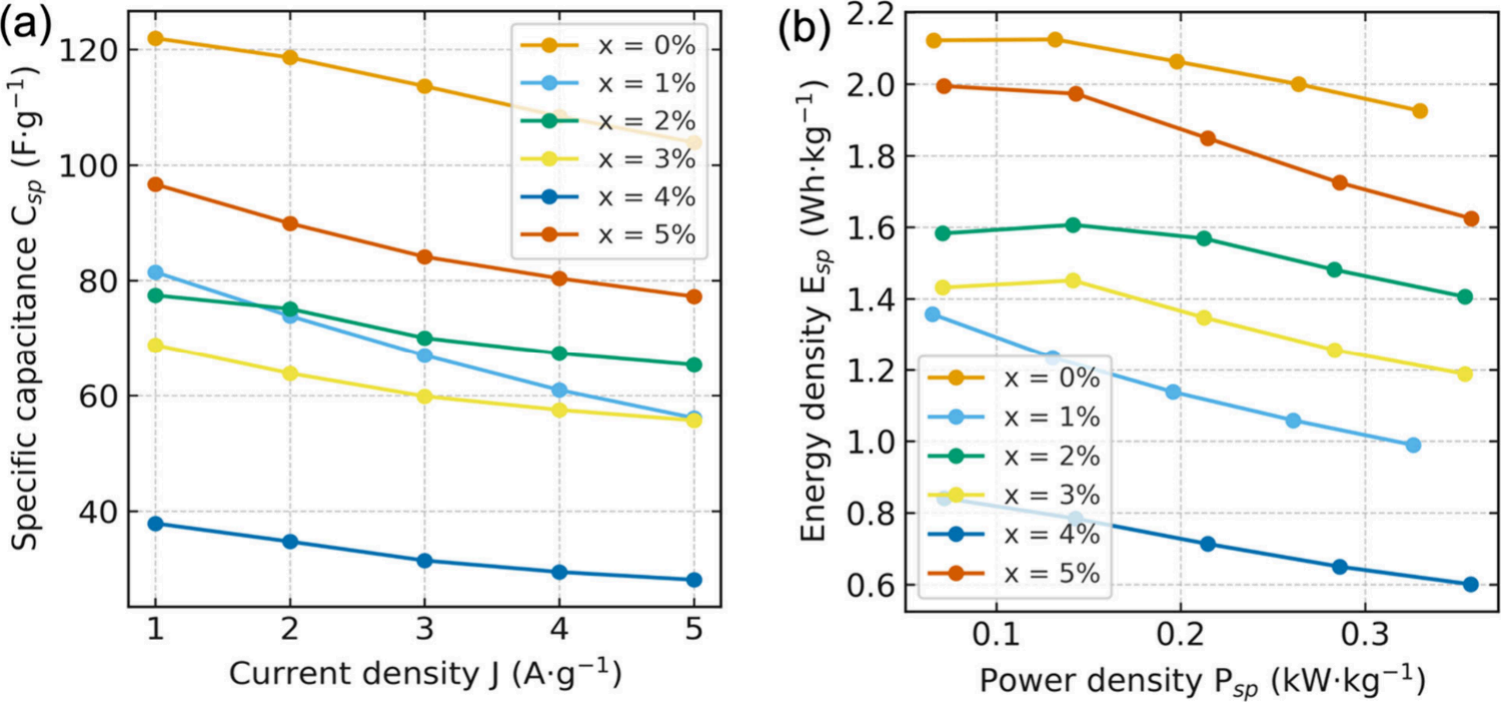
Summary of GCD-derived performance of Ni-doped CuO electrodes (x = 0–5%). (a) Rate capability: specific capacitance *C*_*sp*_ as a function of current density *J* (1–5 *A*·*g* ^–1^), obtained from the R^2^-window linear-discharge fits. Undoped CuO (x = 0%) maintains the highest *C*_*sp*_ over the whole current range. The x = 5% sample shows the second-highest values with a moderate decrease with *J*; x = 2% and x = 3% exhibit intermediate, gently declining *C*_*sp*_; whereas x = 1% drops more strongly with *J* and x = 4% remains lowest across the series. (b) Ragone plot derived from the same GCD data using the working-voltage window. All compositions display the expected trade-off between energy and power density: the x = 0% electrode delivers the largest *E*_*sp*_ across the accessible *P*_*sp*_ range, x = 5% provides the next-best high-power performance, x = 2% and x = 3% show balanced intermediate behavior, while x = 1% and especially x = 4% exhibit comparatively lower figures of merit.

The Ragone plot, constructed from the same GCD data set (Figure 6b), translates these rate characteristics into an energy–power space. Across the accessible power range, the x = 0 electrode occupies the upper envelope, delivering the highest gravimetric energy density (*E*_*sp*_) at a given power density (*P*_*sp*_). The x = 5% composition traces a nearby curve shifted slightly toward higher power, reflecting improved electronic transport relative to intermediate dopings but a smaller effective charge-storage reservoir. The x = 2% and 3% electrodes populate a balanced midregion of the Ragone plane, exhibiting superior energy retention under increasing power compared to x = 1% and x = 4%, and therefore representing the most favorable compromise between capacity and rate capability. In contrast, x = 1% and especially x = 4% remain confined to the lower-energy portion of the diagram, indicating kinetic penalties associated with under- and overdoping. Together with the EIS-derived transport parameters and R2WLD slopes (Tables S5 and S6, Supporting Information), these results demonstrate that Ni incorporation modulates the interplay between electronic percolation and ionic diffusion in a strongly nonlinear manner. It should be noted that the absolute gravimetric energy densities reported here are obtained from three-electrode measurements in aqueous 3 M KOH under a stability-limited voltage window and are therefore inherently constrained by the quadratic dependence of stored energy on voltage (*E*∝ *V* ^2^). The values should therefore be interpreted primarily as electrode-level metrics for comparing intrinsic electrochemical kinetics across Ni compositions under identical conditions. As summarized in Table S7 (Supporting Information), significantly higher energy densities in CuO-based systems are typically achieved only when the device voltage is expanded through asymmetric cell architectures (∼1.5–1.8 V) and/or when conductive hybrid scaffolds (carbon, polymer, or hierarchical core–shell structures) are introduced. In this context, the present Ni–CuO electrodes remain practically relevant as low-cost, scalable pseudocapacitive materials optimized for high-power operation, whose device-level energy density could be further increased through asymmetric supercapacitor design without altering the underlying redox chemistry. Future work will focus on assembling asymmetric supercapacitor devices to evaluate the achievable device-level energy density of the Ni–CuO system.

The observed optimum around x ≈ 2–3% can be rationalized in terms of defect-engineered transport. At moderate substitution, Ni^2+^ replacing Cu^2+^ perturbs the Cu–O network just enough to introduce additional carriers and shallow defect states, enhance Ni/Cu–O orbital overlap, and lower the activation barrier for small-polaron hopping, thereby supporting rapid pseudocapacitive exchange while preserving continuous diffusion pathways.^60^ Beyond this window, accumulated lattice strain, defect clustering, and possible NiO_*x*_–rich regions introduce scattering centers and dead volume, which fragment both the electronic and ionic networks, as reflected in the depressed *C*_*sp*_ and narrowed linear windows for x = 4%. At x = 5%, localized Ni-rich regions appear sufficiently interconnected to re-establish long-range conduction under galvanostatic drive, partially restoring rate capability but not fully recovering the energy density of pristine CuO. This nonmonotonic behavior is characteristic of percolation-type transitions in doped transition-metal oxides, where improved band overlap competes with dopant-induced disorder.^61^ The combined analysis of Figure 6 and Figures S5–S11 (Supporting Information) demonstrates that the R2WLD framework provides a rigorous, composition-resolved benchmark for pseudocapacitive CuO. Algorithmic window selection ensures that rate comparisons are based on equivalently linear segments of the discharge, making the extracted *C*_*sp*_, *E*_*sp*_, and *P*_*sp*_ directly comparable across Ni contents. Within this framework, Ni concentrations of 2–3% emerge as an optimal defect-engineering window that balances conductivity and diffusion, whereas heavier doping (4–5%) reveals the onset of disorder-dominated transport with partial recovery via Ni-rich percolation paths.

To explicitly quantify long-term durability, we carried out continuous GCD cycling for 5000 cycles at 2 *A*·*g* ^–1^ in 3 M KOH. The preservation of reproducible charge–discharge waveforms in the early cycles (Figure S12a–f) indicates that the faradaic response remains highly reversible during extended operation. As summarized in Figure S12g–l, all compositions (x = 0–5%) exhibit stable cycling without progressive polarization drift, and the capacitance retention increases steadily to 147.64% (x = 0), 130.88% (x = 1%), 126.59% (x = 2%), 139.19% (x = 3%), 152.45% (x = 4%), and 148.74% (x = 5%) at 5000 cycles. Retention values exceeding 100% are commonly attributed to electrochemical “activation” rather than degradation. In oxide-based pseudocapacitive electrodes, the initial capacitance (*C*_0_) may underestimate the steady-state response because repeated cycling can promote electrolyte wetting of the porous structure, progressive exposure of redox-active sites, and mild surface reconstruction toward more electroactive Cu-based (oxy)­hydroxide species. Notably, the stronger activation trends at higher Ni contents (x ≈ 4–5%) suggest that Ni incorporation may facilitate sustained utilization of redox-active sites and improved electrolyte accessibility during prolonged cycling.

### 4.3 EIS Analysis Methodology and Equivalent-Circuit Formalism

Electrochemical impedance spectroscopy (EIS) offers a powerful macroscopic tool for probing charge transport, defect chemistry, and polarization dynamics in transition-metal oxide electrodes. In copper­(II) oxide (CuO), electronic transport has frequently been interpreted in terms of thermally activated hopping processes, often described within the small-polaron framework involving Cu^2+^/Cu^3+^ valence fluctuations.^62^ In the present work, this transport picture is invoked only as a qualitative framework consistent with the observed room-temperature electrochemical trends and impedance characteristics. Direct verification of a small-polaron hopping mechanism and extraction of activation energies would require temperature-dependent conductivity measurements, which are beyond the scope of the present study.

The intrinsic transport behavior of CuO is further influenced by native defects such as copper vacancies and interstitial oxygen, which introduce acceptor-like states near the valence-band maximum. The incorporation of Ni^2+^ into the CuO lattice adds additional complexity through dopant-induced lattice strain, defect–dopant interactions, and modified orbital overlap. Consequently, Ni substitution can reshape the local density of states, alter charge-carrier concentration, and modify the effective transport landscape for charge carriers. These structural and electronic perturbations manifest in the impedance spectra as coupled resistive and capacitive responses across the frequency domain. In disordered transition-metal oxides, charge transport mediated by localized carriers often produces impedance spectra characterized by depressed semicircles and frequency-dependent relaxation processes. Such behavior is commonly associated with hopping-type transport between mixed-valence sites and distributed defect states, which provides a qualitative explanation for the nonideal capacitive response and dispersion captured by the CPE elements in the equivalent-circuit model.

This study examines Ni-doped CuO nanoparticles (x = 0 to 5%) through the lens of EIS, interpreted via an equivalent circuit model designed to reflect the underlying physical processes. Following a comprehensive fitting procedure, the extracted parameters, including series resistance (*R*_*s*_), charge-transfer resistance (*R*_*ct*_), constant-phase element coefficients (*Q*, α), and derived quantities such as relaxation frequency (*f*_max_) and effective capacitance (*C*_*eff*_), are correlated to the evolving microstructure and defect landscape. The analysis proceeds in order of increasing frequency-space resolution, beginning with the Nyquist formalism, followed by Bode magnitude and phase plots, and concluding with a residual analysis. A detailed description of EIS Experimental Modeling and Equivalent Circuit Considerations was shown in Note S3 (Supporting Information).

#### 4.3.1 Impedance Semicircles and Structural Signatures: Nyquist Analysis

The Nyquist response separates naturally into an interfacial (high–mid frequency) regime and a transport-limited (low-frequency) regime. The high-frequency intercept on the real axis corresponds to the series resistance *R*_*s*_, which includes the electrolyte resistance together with intrinsic and contact resistances of the electrode. The subsequent depressed semicircle originates from interfacial charge-transfer and polarization processes and is described by *R*_*ct*_ in parallel with a nonideal capacitive element. The diameter of this arc therefore reflects the magnitude of *R*_*ct*_, while its depression indicates a distribution of relaxation times arising from surface roughness, grain boundaries, and heterogeneous active sites; this behavior is captured by *CPE*_1_ in the equivalent-circuit model. At lower frequencies, the spectrum transitions to an inclined tail that is characteristic of ion-transport limitations and diffusion polarization within the porous electrode structure. This diffusion-controlled response is commonly associated with Warburg-type impedance and is represented phenomenologically by *CPE*_2_. Consequently, variations in semicircle diameter primarily quantify interfacial charge-transfer kinetics, whereas changes in the slope of the low-frequency tail provide insight into ion-transport constraints and their evolution with Ni incorporation. All spectra were fitted using the same equivalent-circuit model and fitting protocol to ensure consistent comparison of transport and interfacial parameters across the Ni-doping series.

Consistent with this interpretation, Figure 7a–f shows that all compositions (x = 0–5%) exhibit a depressed arc followed by an inclined low-frequency segment, indicating coupled interfacial polarization and ion-transport contributions. The reduced curvature across the high-to-mid frequency range confirms that the capacitive response is nonideal, justifying the use of constant-phase elements to account for heterogeneous time constants in nanoparticulate oxides. The emergence and persistence of the low-frequency tail further indicate that electrolyte penetration and ion diffusion through the porous microstructure contribute measurably to the overall impedance, particularly under conditions where interfacial charge transfer is accelerated, and transport becomes comparatively rate-limiting.

**Figure 7:**
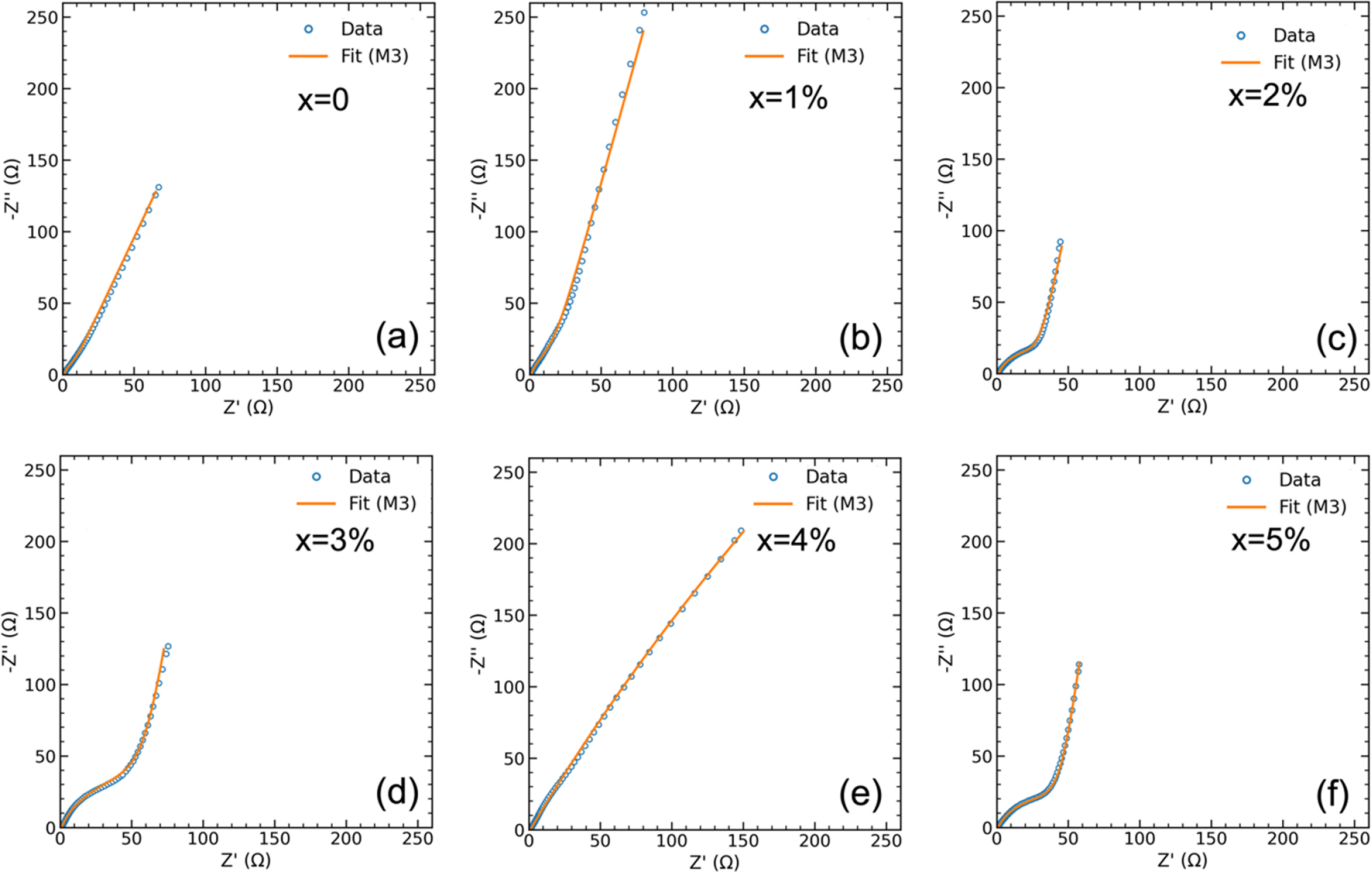
Nyquist plots of Ni-doped CuO nanoparticles (x = 0–5%) obtained from electrochemical impedance spectroscopy (EIS), overlaid with fitted curves using an equivalent circuit model. (a)–(f) show the impedance spectra for x = 0 to 5% Ni doping, respectively. The high-to-mid frequency depressed semicircle reflects interfacial charge-transfer resistance (*R*_*ct*_) in parallel with nonideal capacitive behavior (CPE), while the inclined low-frequency tail is associated with diffusion/ion-transport limitations (Warburg-type behavior) and intergranular polarization. All panels are plotted using identical *Z*′ and −*Z*″ axis limits (equal scaling) to enable direct comparison across compositions. The experimental data (blue open circles) are fitted with the equivalent circuit model (orange solid line), and the evolution of arc diameter/curvature highlights the impact of Ni incorporation on electrical transport and microstructural uniformity; a pronounced increase in semicircle diameter at x = 3% indicates a maximum in *R*_*ct*_, revealing dominant interfacial trapping and dielectric polarization.

Quantitatively, the diameter of the depressed arc in the Nyquist plot is proportional to the charge-transfer resistance *R*_*ct*_, which exhibits a nonmonotonic dependence on Ni concentration rather than a systematic decrease. From Table 1, pristine CuO (x = 0) shows a moderate *R*_*ct*_ value of 4.28 Ω, indicating relatively efficient interfacial charge transport. Upon initial Ni incorporation, *R*_*ct*_increases significantly and reaches a maximum of 64.87 Ω at x = 3%, reflecting strong suppression of interfacial charge transfer. This behavior suggests that intermediate Ni doping introduces substantial lattice distortion, defect trapping, or energy barriers at the electrode–electrolyte interface, thereby hindering carrier exchange. At higher doping levels (x = 4–5%), *R*_*ct*_ decreases again to 35.56 Ω and 44.74 Ω, respectively, implying partial recovery of transport pathways, possibly due to dopant-induced percolation or formation of Ni-rich conductive bridges. These results indicate that the lowest charge-transfer resistance is observed for undoped CuO, while moderate Ni doping initially deteriorates rather than improves interfacial kinetics.

**Table 1:** Electrochemical Impedance Spectroscopy (EIS) Fitting Parameters for Ni-Doped CuO Nanoparticles (x = 0–5% Ni)^a^

x (%)	*R*_*s*_ (Ω)	L (× 10^–7^ *F*)	*R*_*ct*_ (Ω)	α_1_	*Q*_1_ (*S*·*s* ^α_1_^)	α_2_	*Q*_2_ (*S*·*s* ^α_2_^)	*f*_max_ (*Hz*)	*C*_*eff*_ (*F*)	*RMSE* (Ω)	*R* ^2^
0	1.069	1.41	4.284	0.8502	0.00443	0.7162	0.00448	3.1479	0.002829	0.3924	0.99966
1	1.072	1.27	9.217	0.8098	0.00244	0.8013	0.00244	4.0307	0.001320	1.7262	0.99729
2	1.334	2.12	25.396	0.7601	0.00175	0.8385	0.00668	2.8251	0.000878	0.5706	0.99842
3	1.044	1.22	64.869	0.7452	0.00154	0.9552	0.00471	1.1032	0.000941	0.7537	0.99884
4	1.132	2.01	35.555	0.7124	0.00481	0.7277	0.00275	0.6889	0.003153	2.5994	0.99496
5	0.920	1.94	44.744	0.7046	0.00136	0.9199	0.00494	3.1709	0.000561	0.2824	0.99980

a The table summarizes the fitted equivalent circuit parameters based on the equivalent circuit model. Parameters include series resistance (Rs), inductance (L), charge-transfer resistance (Rct), and constant-phase element values (Q1, α1 for high-frequency arc; Q2, α2 for low-frequency tail). Also included are the characteristic frequency (fmax), effective capacitance (Ceff), and goodness-of-fit (R2). These values elucidate the doping-dependent evolution of interfacial and bulk charge transport behavior.

Simultaneously, the pseudocapacitance coefficient *Q*_1_ decreases with doping from 0.00443 *S*·*s* ^α^ at x = 0% to 0.00154 *S*·*s* ^α^ at x = 3%, reflecting a reduction in effective capacitive storage at the interface. In contrast, the low-frequency element *Q*_2_ increases significantly at x = 3%, suggesting enhanced bulk polarization or slow diffusive accumulation in the low-frequency regime. This divergence between high- and low-frequency capacitance components indicates that charge storage progressively shifts from interfacial to bulk-controlled processes upon Ni incorporation.

Interestingly, the constant-phase exponent *α*_*1*_ increases monotonically from 0.8502 (x = 0) to 0.9552 (x = 3%), approaching ideal capacitive behavior (α = 1). This flattening of the Cole–Cole arc suggests that Ni doping leads to a more homogeneous relaxation landscape and reduced nanoscale fractal roughness. In solid-state terms, the increase in α is indicative of improved long-range ordering and coherent hopping channels for small polarons.^62^ The key structural and electrical transformation culminates at x = 3%, where the system achieves. At this point, *R*_*s*_ is at a local minimum (1.044 Ω), but *R*_*ct*_ reaches its maximum value (64.87 Ω), indicating strong charge-transfer suppression rather than enhancement. Similarly, *Q*_1_ decreases to its minimum value, while *Q*_2_ is maximized, and *α*_*1*_ reaches a near-ideal value (0.9552). This set of conditions rules out a percolation-driven conduction transition and instead indicates a defect-dominated transport regime in which deep trap states hinder charge mobility rather than enhance it.^63^ At higher doping levels (x = 4% and 5%), the Nyquist arcs begin to broaden and slightly increase in diameter. *R*_*ct*_ from its maximum value to 35.56 Ω and 44.74 Ω for x = 4% and x = 5%, respectively, indicating partial recovery of charge transport pathways. Concurrently, *α*_*1*_ decreases, reflecting renewed heterogeneity, likely due to the formation of defect complexes and nonconducting NiO-like regions that disrupt previously coherent pathways.

From a theoretical standpoint, these Nyquist trends align with the effective medium approximation for 3D percolative systems, where the conductivity σ follows a scaling law:

6 $$\sigma \propto {\left(p-p_{c}\right)}^{t}$$

Here, *p* is the dopant concentration, *p*_*c*_ the percolation threshold, and *t* ≈ 1.5–2 for three-dimensional systems. The observed peak in *R*_*ct*_ at x = 3% maximum transport hindrance rather than conductivity enhancement, indicating that Ni substitution does not activate conductive clusters but instead deepens interfacial energy barriers.^64^

^64^

#### 4.3.2 Frequency-Domain Behavior: Bode Magnitude and Phase Response

The Bode representation of impedance magnitude (*|Z|* vs frequency) reveals frequency-dependent transitions among bulk conduction, interfacial charge transfer, and low-frequency capacitive accumulation. As shown in Figure S13a–f (Supporting Information), all samples demonstrate three canonical regions across the frequency spectrum: (i) a low-frequency plateau corresponding to dominant interfacial polarization and faradaic processes, (ii) a midfrequency dispersion region associated with constant-phase-element (CPE) behavior due to surface heterogeneity, and (iii) a high-frequency decay governed by bulk charge transport and minor instrumental inductive effects.

The progression of these regions with Ni doping provides insight into the impact of substitution on transport dynamics. Notably, the transition between the midfrequency and low-frequency regions shifts toward higher frequencies as the Ni concentration increases. For undoped CuO (x = 0%), the transition occurs near 450 Hz. At x = 3%, this crossover shifts rightward to nearly 1100 Hz, reflecting enhanced charge-transfer kinetics and a substantial reduction in relaxation time τ.^65^ This frequency shift is corroborated by the derived relaxation frequencies (*f*_max_) listed in Table 1, which decreases from 3.15 Hz (x = 0%) to 1.10 Hz (x = 3%), this apparent reduction does not contradict the Bode-plot trend, because *f*_max_ corresponds to the peak of the relaxation process within the fitted circuit model, whereas the Bode crossover reflects the frequency at which diffusion-dominated impedance transitions to charge-transfer-controlled behavior. Both analyses consistently indicate improved interfacial kinetics at x = 3%, despite arising from different physical formalisms.

Concomitantly, the effective capacitance (*C*_*eff*_) extracted at the *f*_max_ point decreases from 2.83 mF (x = 0) to 0.94 mF (x = 3%), indicating reduced bulk charge accumulation and faster discharge capability. This trend signifies a shift from diffusion-dominated storage toward surface-controlled pseudocapacitive behavior near the optimal doping level. At higher Ni content (x = 5%), *C*_*eff*_ further decreases to 0.56 mF, consistent with structural disorder, carrier localization, and partial blockade of charge-transport pathways.

The dramatic change in impedance slope across the midfrequency range provides further support for this interpretation. For x = 3%, the steepest decline in *|Z|* corresponds to a rapidly evolving charge-transfer regime, indicating minimal resistive hindrance and faster transition to capacitive behavior. In contrast, at x = 5%, this slope flattens again, suggesting the reintroduction of diffusion limitations and potential defect trapping.

#### 4.3.3 Phase Angle Behavior and Dielectric Homogeneity

The phase angle (θ) as a function of frequency, as shown in Figure S14 (Supporting Information), offers complementary insights into dielectric relaxation and interface uniformity. Each curve reveals one or more maxima in phase lag, which arise due to the interplay of resistive and capacitive components within the equivalent circuit model. These maxima are particularly sensitive to the exponent α of the constant-phase elements (CPEs), which encapsulate the dispersion of relaxation times.

For x = 0, the phase maximum occurs around −58°, reflecting a broad distribution of time constants arising from structural disorder, grain boundary irregularities, and inhomogeneous doping. As the Ni content increases, the maximum sharpens and shifts to higher frequencies, reaching a maximum of approximately −72° at x = 3%. This behavior correlates directly with a pronounced increase in the low-frequency CPE exponent α_2_ from 0.7162 (x = 0) to 0.9552 (x = 3%), approaching the ideal capacitive limit (α = 1) (Table 1). In contrast, α_1_ decreases with doping (from 0.8502 to 0.7452 at x = 3%), demonstrating that dielectric improvement arises from interfacial charge accumulation rather than bulk transport uniformity. This sharpening of the phase response reflects enhanced dielectric coherence and a narrowing of the relaxation time distribution, signatures of improved electrode–electrolyte coupling and reduced deep-trap density.^62^ Thus, dielectric homogeneity at x = 3% is governed by α_2_, confirming that the system transitions into an interface-dominated storage regime. From a solid-state standpoint, α_2_ can be considered a measure of order in the conduction pathways: values approaching 1 indicate near-ideal Debye-like behavior, while lower values suggest a fractal or percolative transport regime. Interestingly, beyond the optimal doping level, the phase peak broadens again and shifts back to lower frequencies. At x = 5%, α_2_ decreases to 0.9199, and the phase maximum becomes less distinct. This reversal suggests the onset of dopant clustering and defect-induced disorder, likely associated with Ni agglomeration or the formation of secondary phases such as NiO. These defects introduce deeper trap states that increase recombination and widen the distribution of dielectric response times.

The dual CPE model used in the equivalent circuit fitting captures this behavior with good fidelity, as indicated by the close alignment between fitted and experimental phase curves across all doping levels. The Bode phase plots therefore reveal that Ni doping initially increases dielectric dispersion and interfacial polarization rather than homogenizing the dielectric response, with the strongest relaxation-time broadening occurring at x = 3%. While x = 3% corresponds to maximal electrochemical activity, it does not represent the most uniform dielectric state; instead, undoped CuO (x = 0%) exhibits the most coherent relaxation behavior across frequency, whereas higher doping (x = 4–5%) shows partial dielectric recovery due to Ni-rich conduction pathways.

#### 4.3.4 Fitting Residuals and Model Robustness

To validate the accuracy of the equivalent circuit modeling, Figure S15 (Supporting Information) presents the residual plots for each doping concentration, which capture the difference between the experimental data and the fitted model for both the real (*Z*′) and imaginary (*Z*″) components. Across all doping levels (x = 0–5%), the residuals remain largely within ± 1 Ω, confirming a high degree of fit accuracy and statistical consistency. In the undoped sample (x = 0), small deviations are observed primarily at low frequencies, where capacitive and diffusive effects dominate. These residuals diminish significantly with the introduction of Ni, particularly at x = 3%, where the model reproduces both *Z*′ and *Z*″ components with minimal deviation. The improved fit at this doping level further substantiates the previously identified optimum in transport behavior, surface homogeneity, and interfacial kinetics. Minor increases in residual amplitude are detected again at x = 4% and x = 5%, aligning with the nonmonotonic behavior seen in *R*_*ct*_, *Q*, and α parameters. These discrepancies likely reflect the inability of the idealized equivalent circuit to fully capture the complexity of emerging defects at higher dopant levels. Such complexity may include multiphase formation, increased grain boundary resistance, or nonlinear diffusion phenomena that deviate from the assumptions of CPE-based modeling.^63^

From a modeling standpoint, the low residuals across most of the frequency spectrum validate the choice of the *R*_*s*_ + *L* + (*R*_*ct*_∥*CPE*_1_) + *CPE*_2_ configuration, which captures the essential physics of charge-transfer, bulk conduction, and interface polarization in a distributed system. The use of two CPE elements, rather than simple capacitors, reflects the inherent heterogeneity of solid-state materials and the need to account for fractal and distributed relaxation behavior in nanostructured oxides.

#### 4.3.5 Transport Mechanisms in the Ni-Doped CuO Nanoparticles System

Understanding the transport mechanisms in Ni-doped CuO nanoparticles is critical for optimizing their performance in electronic and energy storage applications. Doping with Ni^2+^ significantly influences charge transport, dielectric response, and defect formation within the CuO matrix. These effects stem from the interplay between polaron dynamics, interface modulation, and dopant-induced disorder. To elucidate the underlying mechanisms driving the observed electrical and dielectric behaviors in Ni-doped CuO nanoparticles, the following subsections examine three key aspects: (1) small-polaron hopping and carrier activation, which govern intrinsic electronic conduction pathways; (2) dielectric modulation and interface engineering, which influence capacitive performance and interfacial phenomena; and (3) defect evolution and overdoping effects, which become prominent at higher doping concentrations. Together, these factors provide a comprehensive understanding of the complex interplay between dopant concentration, microstructural changes, and functional transport properties in the system, as discussed below:

1. Small-Polaron Hopping and Carrier Activation: The electronic conduction of CuO is typically dominated by small-polaron hopping, a mechanism wherein charge carriers (holes) localized on Cu^2+^ sites jump to neighboring Cu^3+^ positions. This process is thermally activated and highly sensitive to lattice distortions and electronic correlation. Ni^2+^ doping perturbs this landscape by modifying the Cu–O–Cu superexchange pathways and introducing acceptor levels that modulate the carrier density and mobility.^66^ Contrary to a monotonic enhancement, the fitted EIS parameters show that charge-transfer resistance does not decrease continuously with doping. Instead, *R*_*ct*_ rises sharply and reaches a maximum value of 64.87 Ω at x = 3%, before decreasing again at higher Ni contents (Table 1). This clearly indicates that charge transport is not maximized at x = 3%, but is instead hindered due to carrier localization and defect-induced scattering. Meanwhile, although *Q*_1_ decreases with doping, the low-frequency dispersion exponent α_2_ increases strongly and reaches a maximum value of 0.9552 at x = 3%, indicating a transition toward dielectric uniformity and a narrowing of the relaxation-time distribution. This reveals that the improvement at x = 3% is dielectric in nature rather than conductive, and charge transport at this composition should be characterized as trap-limited polaron hopping rather than coherent conduction.
2. Dielectric Modulation and Interface Engineering: The evolution of the phase angle and capacitance response implies a significant modulation of the dielectric properties of the system with Ni incorporation. The variation of *C*_*eff*_ and *Q* values reflects changes in interfacial polarization and space-charge accumulation rather than simple surface-area effects. The effective capacitance remains in the submilli-farad range and reaches *C*_*eff*_ = 9.41 × 10^–4^ *F* at x = 3%, confirming moderate but measurable enhancement of charge accommodation at the electrode/electrolyte interface. Notably, although Q values increase at certain compositions, the highest *Q*_1_ appears at x = 0% (0.00443 *S*·*s* ^α^), while at x = 3% *Q*_1_ = 0.00154 *S*·*s* ^α^, indicating that improved phase response at x = 3% does not arise from maximum pseudocapacitance but from improved relaxation behavior. The α_1_ exponent at x = 3% (α_1_ = 0.7452), although not ideal, marks a transition toward more coherent dielectric behavior relative to intermediate doping levels. These results indicate that dielectric modification with Ni occurs through the redistribution of interfacial states and relaxation dynamics, rather than simply enhancing the electrical storage magnitude, consistent with lattice polarization and dopant-induced local field effects.^67^
3. Defect Evolution and Overdoping Effects: Beyond x = 3%, a partial reduction in charge-transfer resistance is observed, with *R*_*ct*_ decreasing from 64.87 Ω to 35.56 Ω (x = 4%) and 44.74 Ω (x = 5%), indicating the onset of a competing transport mechanism. While interfacial polarization is maximal at x = 3%, further Ni incorporation appears to increase defect density and introduce secondary conduction pathways through defect-mediated hopping or Ni-rich regions. Simultaneously, the dispersion parameter α_1_ decreases from 0.7452 at x = 3% to 0.7124 and 0.7046 at x = 4% and 5%, respectively, reflecting increasing dielectric heterogeneity and structural disorder. These trends strongly suggest dopant clustering and local phase inhomogeneity, possibly including partial NiO segregation or defect complexes, which disrupt uniform charge relaxation while introducing alternate conduction routes.

This behavior typifies the overdoping regime, where further dopant addition no longer stabilizes electronic transport but instead induces disorder, trap-assisted hopping, and spatially localized conduction paths.^68^ From the perspective of percolation theory, the region beyond x ≈ 3% represents a transition from optimal interfacial control to partial bulk-dominated conduction, in which excess dopants form disordered networks and electrically inhomogeneous domains rather than enhancing uniform conductivity.^69^

Through a systematic EIS investigation of Ni-doped CuO nanoparticles (x = 0–5%), we have elucidated the complex interplay between doping, microstructure, and charge transport from a solid-state physics perspective. Using an equivalent circuit model grounded in physical principles, we extracted resistive, capacitive, and dispersive parameters that map directly onto the system’s evolving defect chemistry and transport behavior. At a doping concentration of x = 3% Ni, the CuO system exhibits maximum interfacial polarization rather than minimum resistance, characterized by the highest charge-transfer resistance (*R*_*ct*_ = 64.87 Ω), the largest effective capacitance (*C*_*eff*_ = 9.41 × 10^–4^ *F*), and a relaxation frequency of approximately 1.10 Hz, signifying the most intense charge accumulation and slowest polarization dynamics. At this composition, the system also shows the highest dielectric coherence, as evidenced by the maximum α_*2*_ value (0.9552) and the strongest phase response, confirming that x = 3% represents the point of optimal interfacial dominance. Beyond this doping level, a partial recovery of bulk transport occurs, indicated by a decrease in *R*_*ct*_ and an increase in dispersion, reflecting the grow of disorder, dopant clustering, and secondary conduction channels. These competing processes degrade interfacial coherence while restoring partial electronic conduction through the bulk.

Collectively, x ≈ 3% marks the percolation threshold for interfacial control, where dielectric coherence, polarization strength, and carrier confinement peak simultaneously. The results provide valuable insight into how aliovalent doping governs interfacial physics in transition-metal oxides, demonstrating that maximum capacitance and impedance do not necessarily coincide with minimum resistance. These findings are directly relevant to the design of materials for supercapacitors, resistive sensors, and thin-film electronic devices, where control of dielectric homogeneity and charge relaxation dynamics is essential.

### 4.4 Structure–Property Relationship: Ni-Induced Lattice Disorder Governs Charge-Storage Regimes

Ni incorporation modifies CuO across crystallographic, vibrational, and interfacial length scales, and these structural changes correlate with a transition in charge-storage kinetics. Rietveld refinement confirms that the tenorite CuO lattice is preserved upon doping, while peak shifts/broadening indicate substitution-induced microstrain; at the highest loading, a minor NiO phase appears, suggesting that the solid-solution limit is approached. Importantly, particle size remains nearly unchanged from x = 0 to 5%, excluding morphology-driven surface-area differences as the primary origin of electrochemical variations. Raman spectra exhibit systematic blue shifts and broadening of the main CuO modes with increasing Ni content, consistent with local symmetry breaking and enhanced disorder/defect scattering. Although Raman hardening implies local bond stiffening, the average unit-cell volume shows a slight increase at x = 5%, indicating that diffraction averages over microstrain, defect chemistry, and the onset of Ni-rich regions at higher substitution levels. Consistently, XPS O 1s reveals a higher-binding-energy shoulder attributable to surface/defect-related oxygen species, which can introduce localized states and modify interfacial transport.

These microstructural signatures map onto a composition-dependent kinetic regime. Capacitive-fraction/*b*-value analyses reveal that capacitive-dominated domains expand up to an intermediate Ni level and then recede slightly, indicating an optimal defect/disorder window that maximizes fast surface-controlled storage before diffusion-limited polarization becomes more prominent. The same trend is reflected in GCD profiles: moderate Ni substitution yields more triangular discharge behavior, whereas heavier substitution partially reverses the rate-form improvement. EIS further supports this nonmonotonic structure–property linkage, where *R*_*ct*_ increases to a maximum at x = 3% and then decreases at higher x, while the CPE exponent approaches more ideal capacitive behavior near x = 3%. Ni doping creates a competition between beneficial defect-enabled pseudocapacitive activation at intermediate substitution and transport penalties associated with interfacial trapping and incipient phase segregation at higher loading, establishing a clear dopant “window” rather than a monotonic improvement pathway.

## 5 Conclusions

This study presents a comprehensive investigation into the structural, morphological, and electrochemical modulation of CuO nanoparticles upon Ni doping, ranging from x = 0–5%. Rietveld refinement and XRD analyses confirmed the retention of the monoclinic CuO phase with minor NiO secondary phases emerging at higher doping levels. A systematic lattice expansion was observed, attributed to the ionic radius mismatch between Ni^2+^ and Cu^2+^. SEM-based machine learning particle sizing revealed negligible differences in grain size distributions across compositions, indicating that particle morphology is not the primary factor influencing electrochemical behavior. Electrochemical analyses, including CV, GCD with *R* ^2^-Window Linear Discharge (R2WLD), and EIS, revealed that moderate Ni doping (2–3%) optimally balances charge-transfer resistance, pseudocapacitance, and kinetic symmetry. Quantum KPCA and Ising model-based regime mapping further supported the emergence of a capacitive-dominated kinetic regime at this optimal doping range.

This work provides a multidimensional framework for understanding how aliovalent substitution in transition metal oxides, specifically Ni in CuO, governs kinetic regimes in pseudocapacitive materials. By integrating advanced tools such as automated image analysis, quantum manifold learning, and algorithmic discharge segmentation, the study establishes a reproducible methodology to disentangle bulk diffusion from surface-controlled kinetics. The application of percolation theory, dielectric analysis, and electronic structure correlation enhances this work beyond phenomenological observation, providing predictive insights for dopant engineering. These findings not only deepen the understanding of defect-driven pseudocapacitance but also propose practical design windows for optimizing doped oxides in energy storage and sensor applications.

Despite its methodological rigor, the study has limitations. First, the analysis is constrained to room-temperature conditions, which prevents quantitative evaluation of thermally activated transport processes (e.g., activation energies associated with hopping/polaron transport or defect annealing) that require temperature-dependent transport measurements. Second, while SEM and EDX provide morphological and compositional insights, 3D tomographic techniques (e.g., FIB-SEM or X-ray nanotomography) can offer deeper clarity on dopant distribution within grain interiors and boundaries. Third, although the Ising model and KPCA uncover rich kinetic landscapes, their applicability to other dopant systems requires further validation. Lastly, two-electrode device-level long-term stability, cycling durability, and thermal robustness of the Ni-doped CuO electrodes remain crucial for real-world deployment and will be addressed in follow-up work.

Building on these findings, future studies should investigate temperature-dependent transport to accurately quantify activation energies. In-situ or operando spectroscopy (e.g., Raman, XPS, or impedance under bias) could capture real-time phase and electronic transitions. Exploring other dopants (e.g., Co, Zn, Mn) using the presented analytical framework may reveal universal trends in aliovalent substitution. Furthermore, coupling density functional theory (DFT) calculations with experimental observations could clarify the electronic origins of observed transitions. Finally, assembling full-cell devices with these optimized materials would test their viability in practical energy storage systems.

This work advances the fundamental and applied understanding of dopant-controlled pseudocapacitive behavior in CuO nanoparticles. It emphasizes the nonlinear, threshold-dependent nature of defect modulation and charge transport, reinforcing the idea that more is not always better in doping strategies. Through a rigorous and multifaceted approach, this study lays the groundwork for intelligent material design, where structural precision and electronic tunability coalesce to meet the demands of next-generation energy technologies.

## Supplementary Material



## Data Availability

Data will be made available on request.

## Notes

Method Note S1 details redox-couple assignment and mechanistic origin of multiple anodic/cathodic peaks in the CV Curves of Ni-doped CuO electrodes; Method Note S2 describing the voltage-heatmap construction and *R* ^2^-optimized linear-window (R2WLD) protocol for GCD capacitance extraction (normalization, window selection, and slope-to-*C*_*sp*_ conversion); Method Note S3 details the EIS experimental modeling and equivalent-circuit fitting workflow (model rationale, CPE formulation, preprocessing, robust nonlinear fitting, bounds, and randomized restarts); Figures S1–S4: Randles–Ševčík linear fits of anodic peak current density vs $\sqrt{\nu }$ (x = 0–5%), potential-dependent distributions of the capacitive coefficient *k*_1_(*E*) and diffusion-controlled coefficient *k*_2_(*E*), and a summary of electrochemical/kinetic metrics (capacitance vs scan rate, *b*-value heatmap, capacitive-fraction heatmap, and Ising-based segmentation); Figure S5: Differential discharge-slope maps (*dV*/*dt*) with white rectangles marking the R^2^-optimized fitting windows for x = 0–5% under *J* = 1–5 *A*·*g* ^–1^; Figures S6–S11: R2WLD GCD fitting panels (x = 0–5%) and compiled rate-capability trends for *J* = 1–5 *A*·*g* ^–1^; Figure S12: Long-term cycling stability of Ni-doped CuO electrodes (x = 0–5%); Figures S13–S15: EIS Bode magnitude plots, Bode phase (phase angle) plots, and corresponding residual plots (*ΔZ*′, *ΔZ*″) versus frequency for x = 0–5%; Tables S1–S6: Rietveld refinement parameters (Table S1), ML-extracted SEM particle statistics (Table S2), EDX elemental composition (Table S3), comparison of experimental Raman-active modes with DFT values (Table S4), compiled GCD fitting parameters (Table S5), and fitting parameters for energy/power-density analysis (Table S6); Benchmark comparison of CuO and CuO-based (including Ni-containing) supercapacitor systems reported in aqueous electrolytes (Table S7) (PDF)
