# Supplementary material for: Redox Modulation and Diffusion Kinetics in Ni-Doped CuO: Insights from Quantum-Inspired Electrochemical Methods
Source: ACS Appl Mater Interfaces. 2026 Apr 21;18(17):24398–418. doi: 10.1021/acsami.5c26218 (PMC13154133; doi:10.1021/acsami.5c26218)
Supplement: Supplementary file 1 [file am5c26218_si_001.pdf]

Supporting Information

Redox Modulation and Diffusion Kinetics in Ni-Doped CuO: Insights from Quantum-Inspired Electrochemical Methods

Tsung-Te Lin[a], Shih-Lung Yu[b], Yi-En Wu[c], Hai Yen Thi Nguyen[b], Wei-Lun Li[d], Yun-Syuan Liang[d], Kai-Hsun Lin[d], Ming-Kang Ho[b, e], Tsu-En Hsu[b, e], Krishtappa Manjunatha[b,*], Yi-Ru Hsu[a], Wei-Che Lo[f], Chia-Liang Cheng[b], B. Daruka Prasad[g], B. K. Monika[g], Hanumanthappa Nagabhushana[h], Meng-Chu Chen[d,*], and Sheng Yun Wu[b,*]

[a] *Department of Mechanical and Systems Engineering, National Atomic Research Institute, Taoyuan 325207, Taiwan*

[b] *Department of Physics, National Dong Hwa University, Hualien 97401, Taiwan*

[c] *Arete Honors Program, National Yang Ming Chiao Tung University, Hsinchu 300093, Taiwan*

[d] *Department of Applied Science, National Taitung University, Taitung 950, Taiwan*

[e] *National Synchrotron Radiation Research Center, Hsinchu 30076, Taiwan*

[f] *Department of Physics, National Tsing Hua University, Hsinchu 30013, Taiwan*

[g] *Department of Physics, BMS Institute of Technology and Management, VTU Belgavi Affiliated, Bangalore 560064, India*

[h] *Prof. C.N.R. Rao Centre for Advanced Materials, Tumkur University, Tumkur 572 103, India*

*Corresponding author: kmanjunatha@gms.ndhu.edu.tw(KM), mchen@nttu.edu.tw (MCC), sywu@mail.ndhu.edu.tw (SYW)

Note S1: Electrochemical Origin of Multiple Redox Peaks in the CV Curves of Ni-Doped CuO Electrodes

The CV measurements were conducted in 3 M KOH using an Ag/AgCl reference electrode within the potential window of 0.0–0.55 V. Under alkaline conditions, CuO-based pseudocapacitance typically involves sequential surface redox reactions of Cu species, while Ni incorporation introduces additional Ni-centered redox processes. As a result, the observed multiple peaks originate from overlapping Cu- and Ni-related redox couples, together with heterogeneity-induced broadening of the electrochemical response.

Specifically, the following redox processes are expected:

(1) Lower-potential redox pair (Cu-centered processes)

Primarily associated with Cu^{2+}/Cu^{+} surface conversion reactions:

$$CuO+H_2O+e^{-}\leftrightarrow Cu(OH)_2 \quad (S1)$$

or equivalently described as:

$$Cu^{2+}+e^{-}\leftrightarrow Cu^{+} \quad (S2)$$

which corresponds to CuO/Cu_2O-like interconversion commonly observed in Cu-based pseudocapacitors.

(2) Higher-potential redox pair (Cu^{3+}-like oxyhydroxide formation)

$$Cu(OH)_2+OH^{-}\leftrightarrow CuOOH+H_2O+e^{-} \quad (S3)$$

(3) Ni-centered redox process introduced by Ni doping

Ni incorporation introduces an additional redox couple that overlaps with the higher-potential Cu process:

$$Ni(OH)_2 + OH^- \leftrightarrow NiOOH + H_2O + e^- \quad (S4)$$

The coexistence of these redox couples leads to partially overlapping anodic and cathodic peaks, producing the multiple peak features observed in the CV curves.

In addition, Ni substitution modifies the electronic structure and defect chemistry of the CuO lattice. Replacement of Cu^{2+} ($3d^9$) by Ni^{2+} ($3d^8$) alters the local crystal field environment and introduces lattice strain and defect states, which broaden the distribution of redox potentials. Consequently, a single ideal redox event becomes distributed over multiple local environments (surface vs. near-surface sites, strained regions, and defect-rich domains), resulting in peak splitting or shoulder formation.

Furthermore, structural analysis indicates the presence of a minor NiO secondary phase (~4 wt.% at x = 5%), which likely resides at grain boundaries or particle surfaces. In alkaline electrolyte, this NiO can be electrochemically expressed through the $Ni(OH)_2$/NiOOH redox couple, further reinforcing the higher-potential oxidation feature.

Figure S1. (a)-(f) Linear fitting of anodic peak current density (I_{peak}) versus square root of scan rate ($\sqrt{\nu}$) for Ni-doped CuO nanoparticles with Ni doping levels of x = 0 to 5%. The strong linear correlation in all cases (R^2 > 0.99) indicates a diffusion-controlled electrochemical process governed by the Randles–Ševčík equation. The variation in slope with increasing Ni content reflects changes in electrochemical kinetics and active surface area resulting from Ni incorporation.

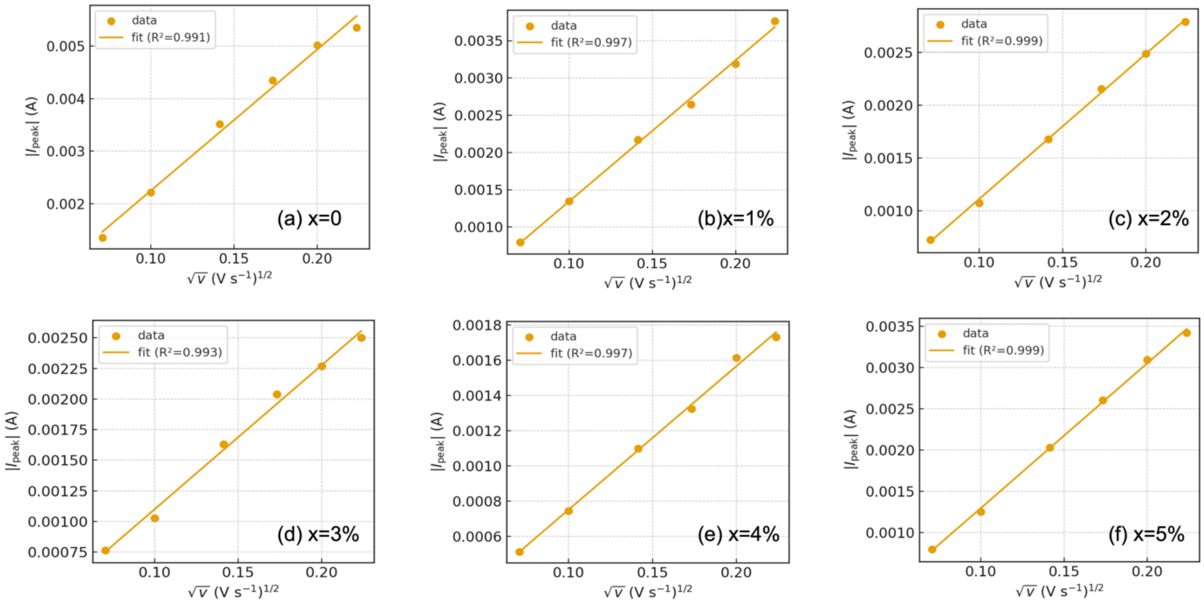

Figure S2. Distribution of the capacitive current contribution coefficient k_1 as a function of potential (E) for Ni-doped CuO nanoparticles at doping levels x = 0 to 5%. (a)–(f) correspond to x = 0 to 5%, respectively. The k_1 values, expressed in $A \cdot (V/s)^{-1}$, were extracted from CV data using the power-law relationship $I = k_1 v + k_2 \sqrt{v}$, isolating the capacitive (surface-controlled) component. Color maps indicate the normalized potential range (red to green) to visualize peak alignment and evolution. Increasing the Ni content leads to notable changes in the capacitive response, suggesting modified charge storage dynamics and enhanced pseudocapacitive behavior at specific doping levels.

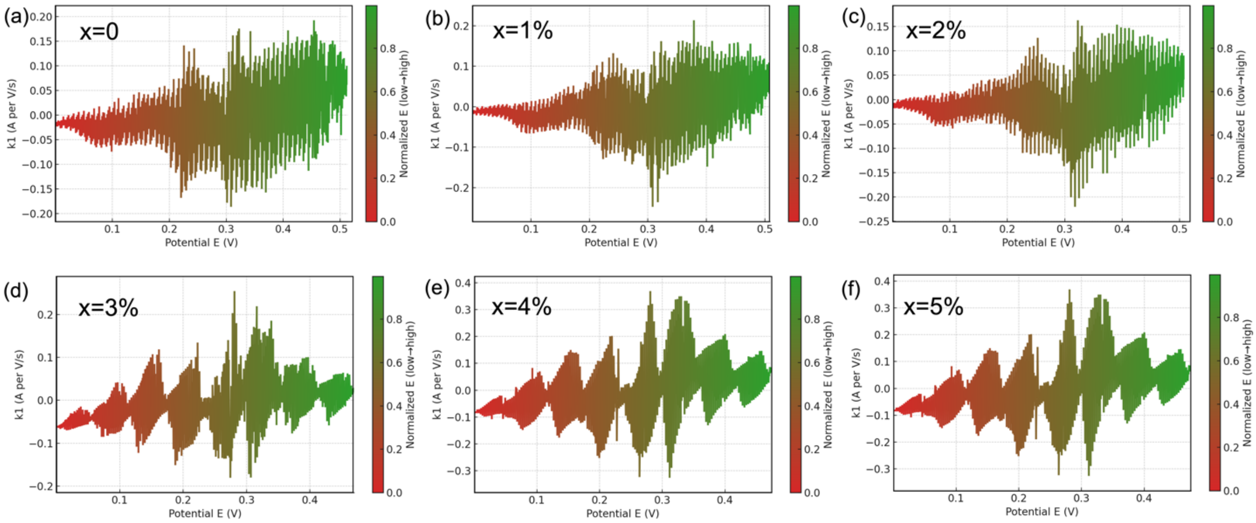

Figure S3. Distribution of the diffusion-controlled current coefficient k_2 as a function of potential (E) for Ni-doped CuO nanoparticles with doping levels ranging from x = 0 to 5%. (a)–(f) represent x = 0 to 5%, respectively. The k_2 values, given in $A \cdot (V/s)^{-1/2}$, are derived from the separation of current response using the equation $I = k_1 v + k_2 \sqrt{v}$, where k_2 characterizes the diffusion-limited process. Color gradients show normalized potential, highlighting how diffusion-related contributions evolve across the electrochemical window. The emergence and growth of sharp peaks with increased Ni doping suggest enhanced redox activity and ion diffusion dynamics in specific potential regions.

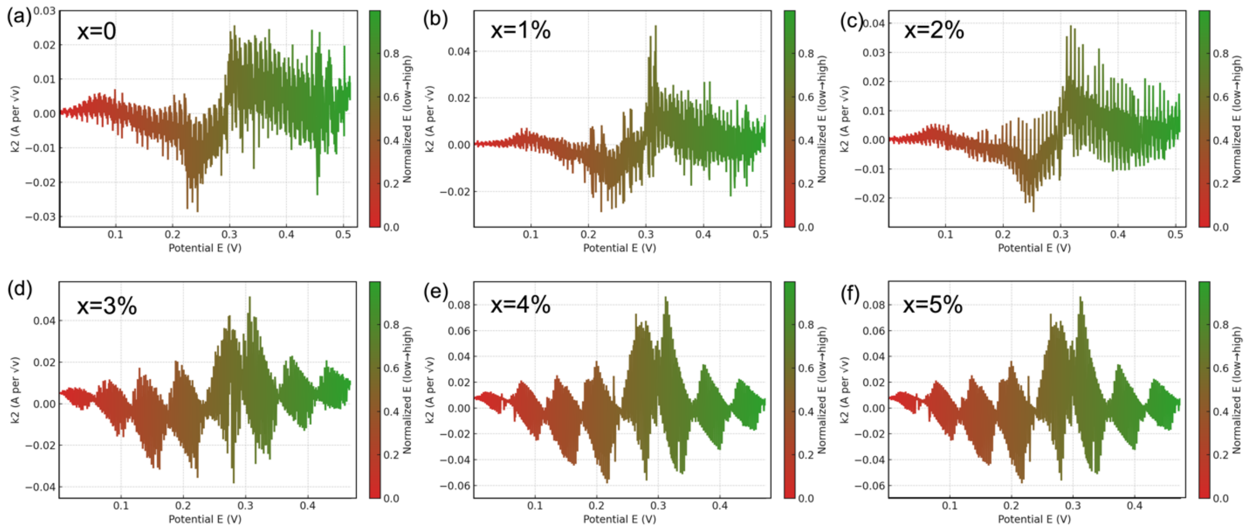

Figure S4. Summary of electrochemical performance and kinetic analysis across Ni-doped CuO nanoparticles with doping levels from x = 0 to 5%. (a) Specific capacitance versus scan rate (5–50 mV/s) for each Ni content. Pristine CuO (x = 0%) exhibits the highest capacitance at low scan rates, while moderate Ni doping (x = 2–3%) balances high capacitance and rate performance. (b) Heatmap of b-values extracted from the power-law relation $I = av^b$, mapped over potential and Ni content. A transition from diffusion- ($b \approx 0.5$) to capacitive-dominated ($b \approx 1$) behavior is observed with increasing doping and potential. (c) Capacitive contribution heatmap at 50 mV/s, showing the fraction of capacitive-controlled current as a function of potential and Ni content. Enhanced capacitive response is evident at intermediate doping levels. (d) Ising-based kinetic regime segmentation map, visualizing binary state transitions (1 = capacitive, 0 = diffusion) across potential and Ni doping. Regions of dominant capacitive behavior expand with doping up to ~3%, then slightly recede, suggesting an optimal doping range for pseudocapacitive enhancement.

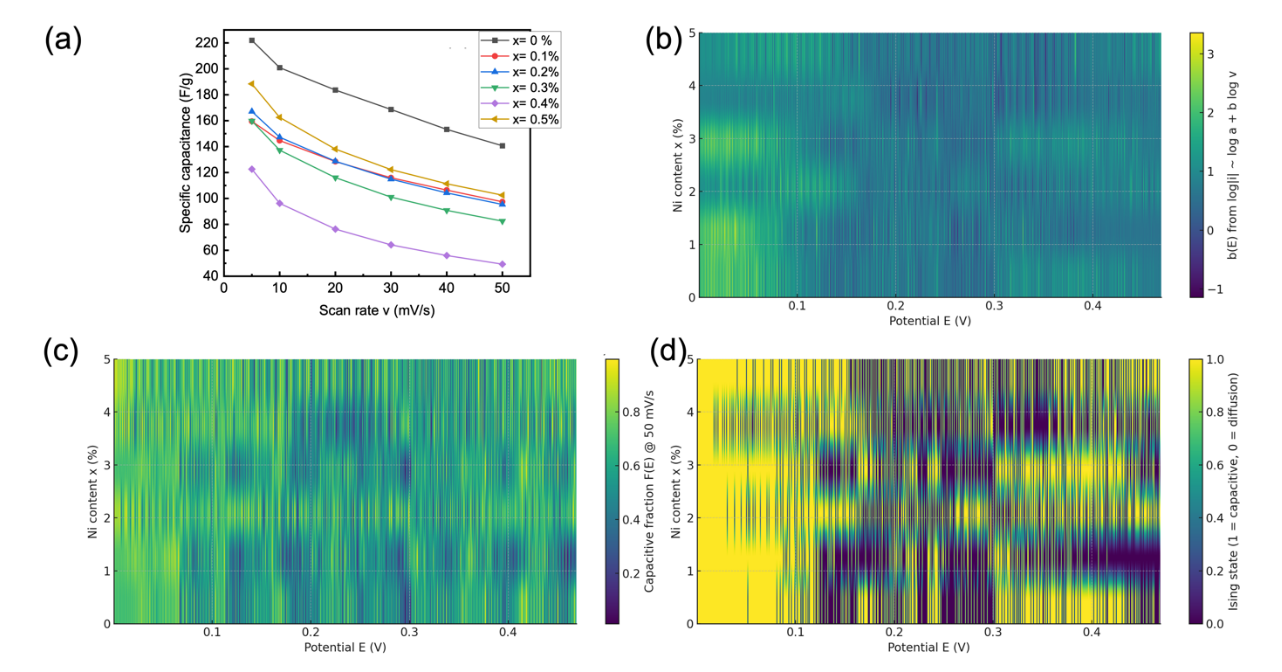

Note S2: The voltage heatmap and the selected fitting windows method

Method description of the Voltage heatmap with selected fitting windows, what it shows, how we build it, and the equations behind the window selection and capacitance extraction.

1. Goal and idea: Given constant current GCD discharge traces $V_J(t)$ at several current densities $J\ (A \cdot g^{-1})$, we want a single figure that (i) compares the entire discharge profile across currents on a common horizontal axis and (ii) marks the linear (capacitive) region used for quantitative slope/Capacitance estimates. The result is a 2D heatmap: x-axis = normalized discharge time [0,1]; y-axis = current density J; color = voltage V.

2. Normalize time across currents: Each current trace has its own discharge duration P_j (from the voltage peak to the end of discharge). For comparability, we define $x \equiv \frac{t}{P_J} \in [0,1],\ P_J = \max(t)$ on the discharge leg. We keep voltages in volts and resample each $V_J(t)$ onto a common uniform grid x_k via 1D interpolation:

$$V_j(x_k) = interp\big(V_J(t), t = x_k P_J\big), k = 1 \dots . N \qquad \text{(S5)}$$

Stacking the rows (one per J) yields a matrix $V \in \mathbb{R}^{M \times N}$ visualized with origin lower so increasing J goes upward.

3. Selecting the linear-fit window (R^2-max): To label the “best” linear segment of the discharge, we scan candidate sub-intervals $[t_a,\ t_b]$ within the discharge leg and perform an ordinary least-squares (OLS) fit

$$V(t) \approx \beta_0 + \beta_1 t,\ \beta = \frac{\sum(t-\bar{t})(V-\bar{V})}{\sum(t-\bar{t})^2},\ \beta_0 = \beta_1 - V_0 t \qquad \text{(S6)}$$

For each candidate window we compute the coefficient of determination

$$R^2 = 1 - \frac{\sum_i (V_i - \hat{V}_i)^2}{\sum_i (V_i - \bar{V}_i)^2} \qquad \text{(S7)}$$

and choose the window that maximizes R^2 subject to a minimum length (to avoid trivial windows). This suppresses the onset of IR-drop and tail curvature, focusing on the near-constant-slope region expected for capacitive behavior.

4. From absolute time to normalized rectangles Let $[t_{start,J}, t_{end,J}]$ be the winning window in seconds for current J. We convert to normalized coordinates for overlay on the heatmap:

$$x_{start,J} = \frac{t_{start,J}}{P_J}, x_{end,J} = \frac{t_{end,J}}{P_J} \quad \text{(S8)}$$

On the heatmap row corresponding to current J we draw a rectangle spanning $[x_{start,J}, x_{end,J}]$ horizontally and a narrow band around J vertically, marking the region used for quantitative fitting.

5. Capacitance from the fitted slope: In ideal constant-current discharge with effective capacitance C_{eff} (per gram), voltage is

$$V(t) = V_o - \frac{I}{C_{eff}} t - IR_{drop} \quad \text{(S9)}$$

so the slope in the linear region is

$$\frac{dV}{dt} = -\frac{I}{C_{eff}} \quad \text{(S10)}$$

If current is expressed as current density $J = I/m(A \cdot g^{-1})$, then the specific capacitance follows directly from the fitted slope $\hat{\beta} = \left|\frac{dV}{dt}\right|$ $(V \cdot s^{-1})$:

$$C_{sp} = \frac{J}{|\hat{\beta}|} \ (F \cdot s^{-1}) \quad \text{(S11)}$$

Reporting C_{sp} vs J (rate capability), alongside the heatmap, connects the visual selection to a quantitative, audit-ready metric.

In short, the voltage heatmap + selected windows provide an intuitive whole-window view while anchoring quantitative analysis to the objectively most linear segment, yielding consistent and reproducible C_{sp} estimates. Importantly, the R2WLD linear fit is designed to describe the most linear mid-discharge segment only; it is not intended as a global model of the entire discharge trace.

Figure S5. (a)-(f) Differential discharge-slope maps, dV/dt (colour scale), for Ni-doped CuO nanoparticles at x=0–5% under GCD, respectively. The x-axis shows the normalized discharge time (0 → 1), and the y-axis the applied current density (J = 1–5 $A \cdot g^{-1}$). The colour bar on the right encodes the instantaneous slope dV/dt ($V \cdot s^{-1}$), spatially uniform, low-contrast regions indicate a nearly constant slope and therefore a quasi-capacitive, linear discharge segment, whereas localized bright features mark departures from ideal linearity. White rectangles denote the R^2-optimized windows used to extract -dV/dt for specific-capacitance calculations. Relative to undoped CuO (x = 0), samples with moderate Ni contents (x ≈ 1–3%) display broad mid-time domains where dV/dt remains nearly invariant across all J, consistent with enhanced rate kinetics and reduced diffusion polarization. At higher substitution levels (x = 4–5%), the slope maps reveal more pronounced early/late-time variations and slightly narrower effective linear windows. These maps provide an objective, visual basis for the window selection employed in the R^2-window linear-discharge analysis discussed in the main text.

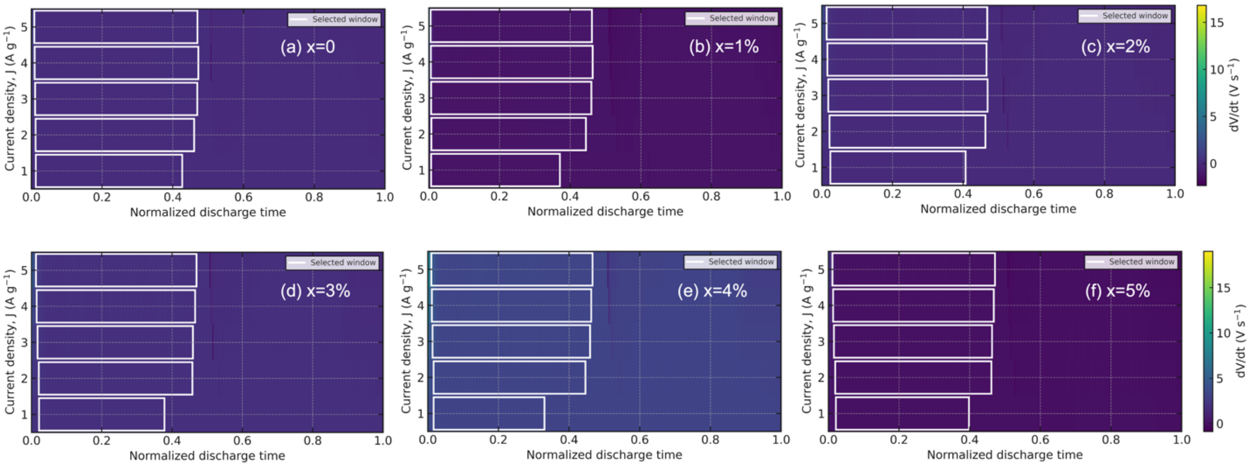

Figure S6. -window linear-discharge (R2WLD) analysis for undoped CuO (x = 0) at J = 1–5 $A \cdot g^{-1}$. (a)–(e) display the GCD discharge curves (orange) together with the 90–10% voltage band (grey shading), the data points selected by the R^2- optimization procedure for linear fitting (cyan), and the corresponding least-squares fit within that window (teal line). For each current density, the fitted slope $|dV/dt|$ is converted into a window-specific capacitance using $C_{sp} = J/|dV/dt|$. (f) summarizes the resulting rate capability as specific capacitance C_{sp} versus current density, demonstrating that the objective window-selection protocol yields internally consistent, audit-ready capacitance values across the full range of J. Note that the teal line is fitted only to the cyan points within the R^2-optimized window; deviations outside this window (initial IR drop and low-voltage tail curvature) are expected and are intentionally excluded from capacitance extraction.

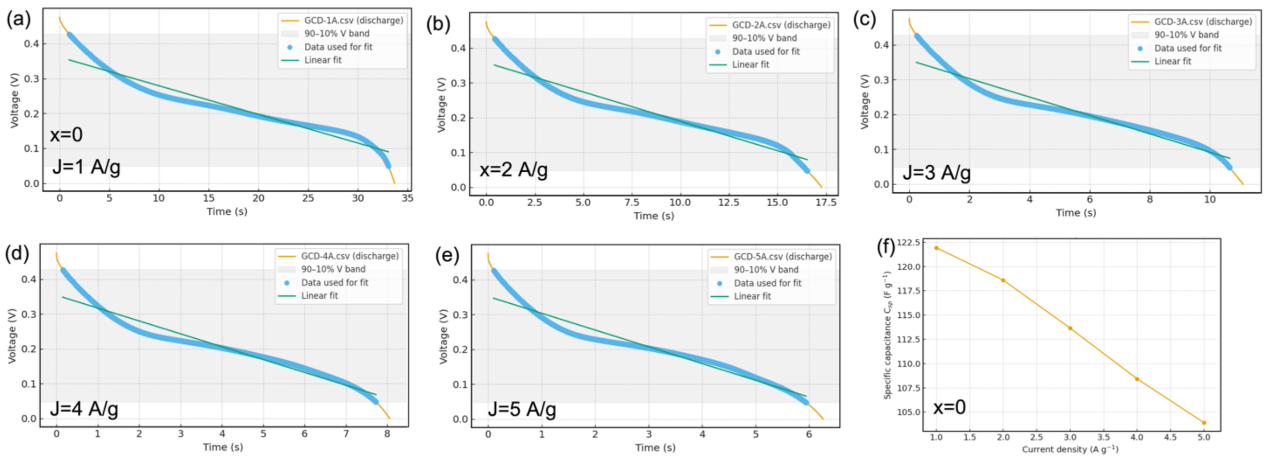

Figure S7. R^2-window linear-discharge (R2WLD) analysis for Ni-doped CuO with x=1% at J = 1–5 $A \cdot g^{-1}$. (a)–(e) display the GCD discharge profiles (orange), the 90–10% voltage band (grey shading), the datapoints selected by the R^2- optimization algorithm for linear fitting (cyan), and the corresponding least-squares fits within those windows (teal lines). For each current density, the fitted slope $|dV/dt|$ is converted to a window-specific capacitance using $C_{sp} = J/|dV/dt|$. (f) compiles the resulting rate capability as specific capacitance C_{sp} versus current density. Compared with undoped CuO, the x=1% electrode retains a well-defined mid-time linear regime across all J, indicating reproducible, kinetically robust capacitance extraction under the R2WLD protocol.

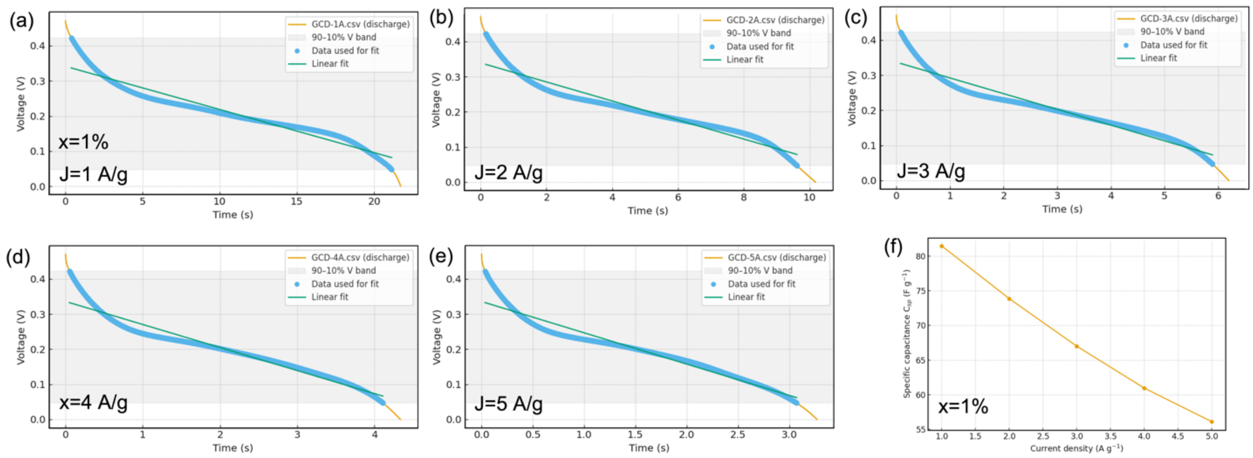

Figure S8. R^2-window linear-discharge (R2WLD) analysis for Ni-doped CuO (x = 2%) at J = 1–5 $A \cdot g^{-1}$. (a)–(e) show the GCD discharge curves (orange), the 90–10% voltage band (grey shading), the datapoints selected by the R^2- optimization routine as the most linear segment (cyan markers), and the corresponding least-squares fits within those windows (teal lines). For each current density, the fitted slope $| dV/dt |$ is converted to a window-specific capacitance using $C_{sp} = J/| dV/dt |$. (f) summarizes the resulting rate capability as specific capacitance C_{sp} versus current density, revealing a gradual decrease from the upper-77 $F \cdot g^{-1}$ at 1 $A \cdot g^{-1}$ to the mid-65 $F \cdot g^{-1}$ at 5 $A \cdot g^{-1}$. The persistence of well-defined mid-time linear regions across all currents indicates efficient charge-transfer kinetics and only modest diffusion-induced curvature at this doping level.

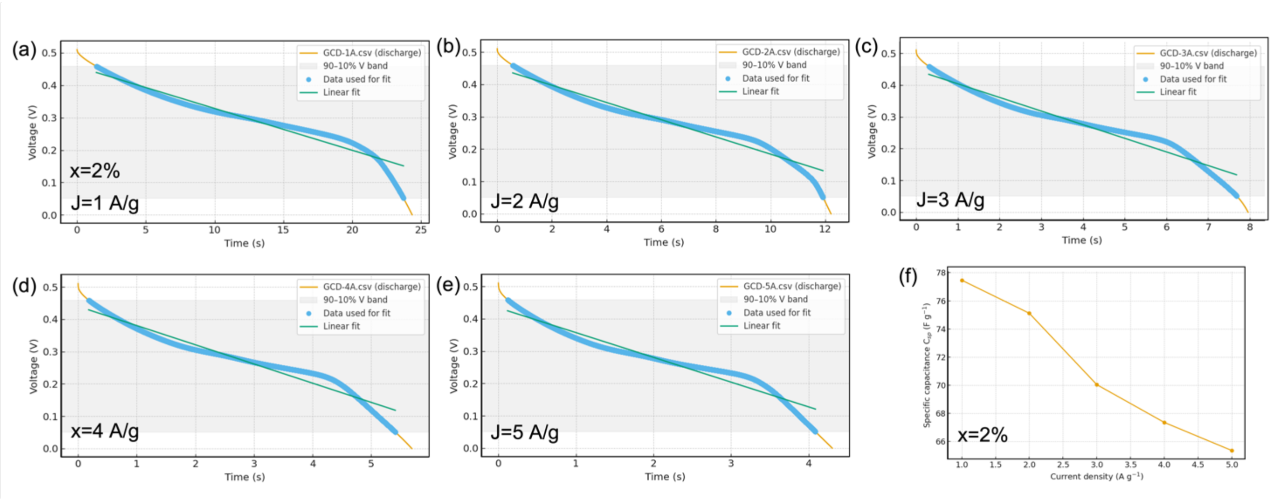

Figure S9. R^2-window linear-discharge (R2WLD) analysis for Ni-doped CuO with x=3% at J = 1–5 $A \cdot g^{-1}$. (a)–(e) present the GCD discharge traces (orange), the 90–10% voltage band used as the search domain (grey shading), the datapoints selected by the R^2- optimization procedure as the most linear segment (cyan markers), and the corresponding least-squares fits within that window (teal lines). For each current density, the fitted slope $|dV/dt|$ is converted into a window-specific capacitance via $C_{sp} = J/|dV/dt|$. (f) compiles the resulting rate capability as specific capacitance C_{sp} versus current density, revealing a moderate decrease from ~69 $F \cdot g^{-1}$ at 1 $A \cdot g^{-1}$ to ~56 $F \cdot g^{-1}$ at 5 $A \cdot g^{-1}$. The persistence of well-defined mid-time linear regions across all J indicates robust charge-transfer kinetics and relatively weak diffusion-induced curvature at this composition.

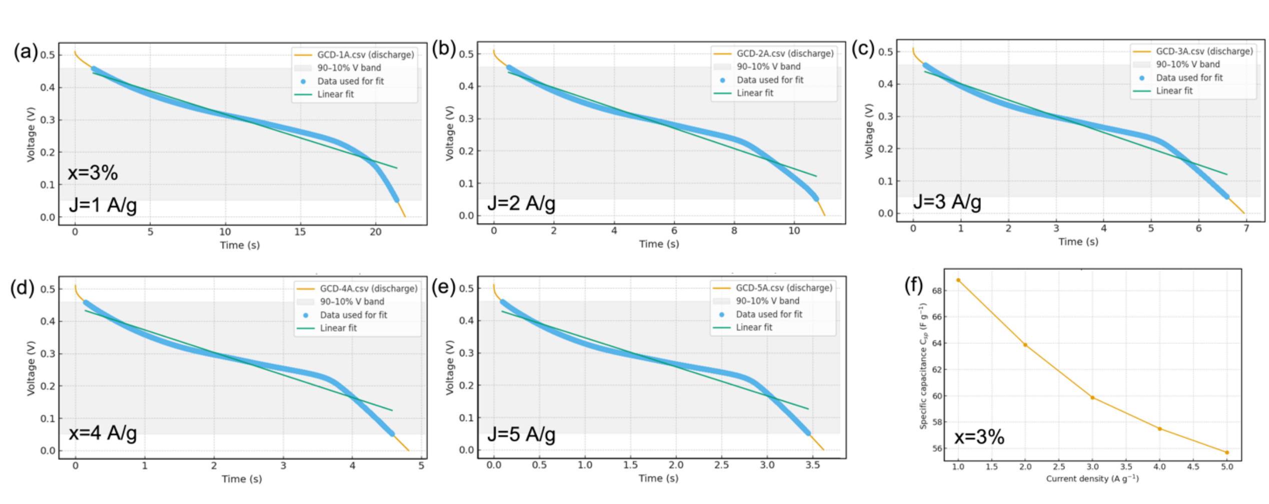

Figure S10. R^2-window linear-discharge (R2WLD) analysis for Ni-doped CuO with x = 4% at J = 1–5 $A \cdot g^{-1}$. (a)–(e) present the GCD discharge traces (orange), the 90–10% voltage band used as the search domain (grey shading), the datapoints selected by the R^2-optimization routine as the most linear segment (cyan markers), and the corresponding least-squares fits within that window (teal lines). For each current density, the fitted slope $|dV/dt|$ is converted into a window-specific capacitance using $C_{sp} = J/|dV/dt|$. (f) compiles the resulting rate capability as specific capacitance C_{sp} versus current density, showing a gradual decrease from~38 $F \cdot g^{-1}$ at 1 $A \cdot g^{-1}$ to ~28 $F \cdot g^{-1}$ at 5 $A \cdot g^{-1}$. Although mid-time linear windows persist over the full current range, they become progressively narrower at higher J, reflecting stronger transport and kinetic limitations at this higher Ni substitution level.

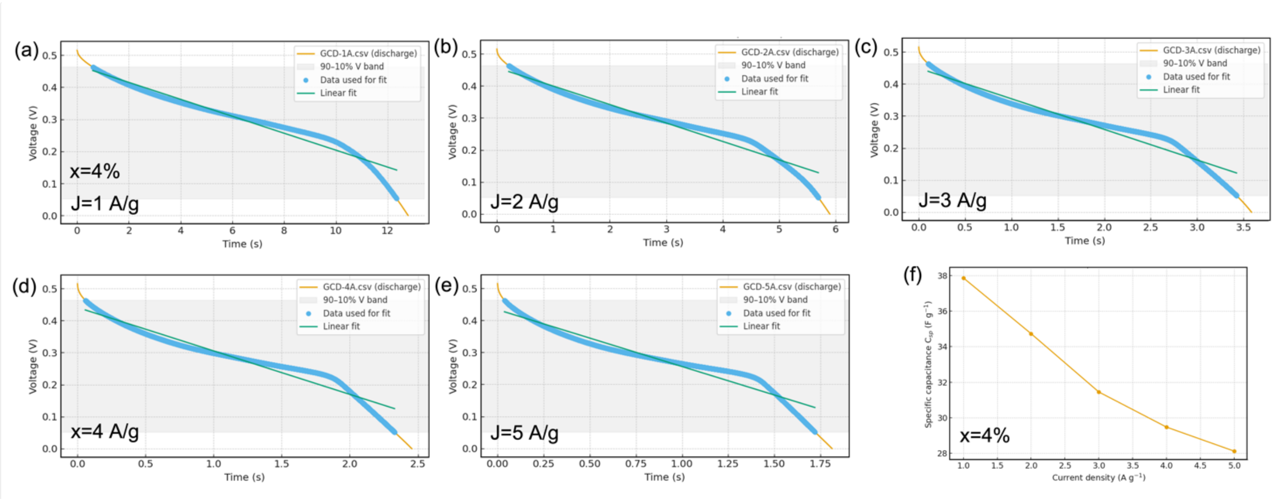

Figure S11. R^2-window linear-discharge (R2WLD) analysis for Ni-doped CuO with x=5% at J = 1–5 $A \cdot g^{-1}$. (a)–(e) show the GCD discharge curves (orange), the 90–10% voltage band used as the search domain (grey shading), the datapoints selected by the R^2- optimization routine as the most linear segment (cyan markers), and the corresponding least-squares fits within those windows (teal lines). For each current density, the fitted slope $|dV/dt|$ is converted into a window-specific capacitance using $C_{sp} = J/|dV/dt|$. (f) summarizes the resulting rate capability as specific capacitance C_{sp} versus current density, revealing a modest decrease from~97 $F \cdot g^{-1}$ at 1 $A \cdot g^{-1}$ to ~77 $F \cdot g^{-1}$ at 5 $A \cdot g^{-1}$. The persistence of well-defined mid-time linear regions across all J indicates that, despite the higher Ni content, the x = 5% electrode maintains relatively robust charge-transfer kinetics with only moderate diffusion-induced curvature.

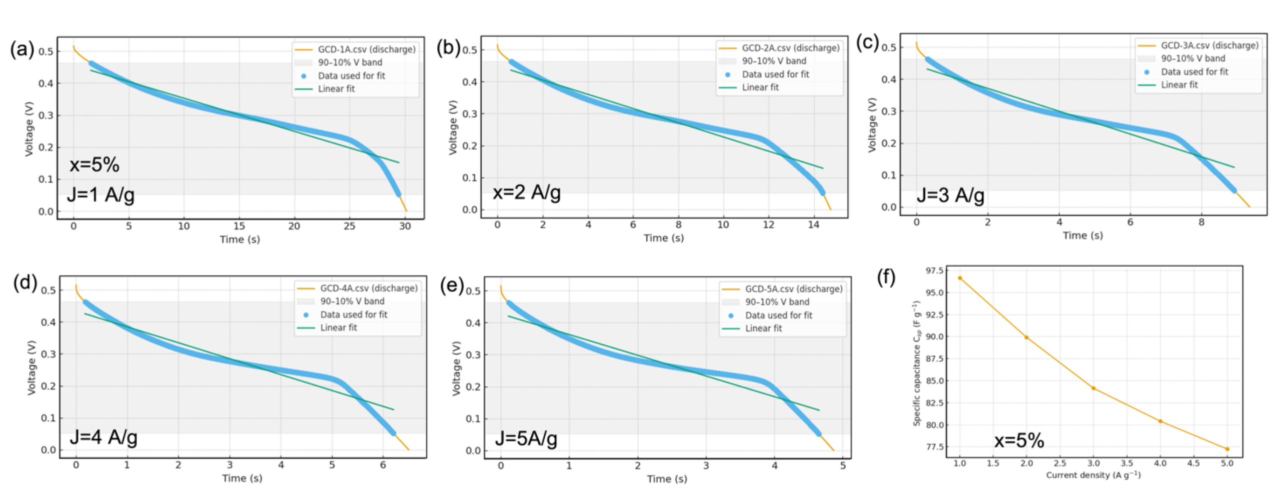

Figure S12. Long-term cycling stability of Ni-doped CuO electrodes (x = 0–5%). (a)–(f) Representative galvanostatic charge–discharge (GCD) profiles of the initial cycles (first 10 cycles) recorded within 0–0.50 V in 3 M KOH, illustrating reproducible charge–discharge behavior for x = 0, 1, 2, 3, 4, and 5% samples. (g)–(l) Capacitance retention ($C/C_0 \times 100\%$) as a function of cycle number during continuous GCD cycling up to 5000 cycles at a fixed current density ($J = 2\ A \cdot g^{-1}$), evidencing stable operation with retention values at 5000 cycles of 147.64% (x = 0), 130.88% (x = 1%), 126.59% (x = 2%), 139.19% (x = 3%), 152.45% (x = 4%), and 148.74% (x = 5%).

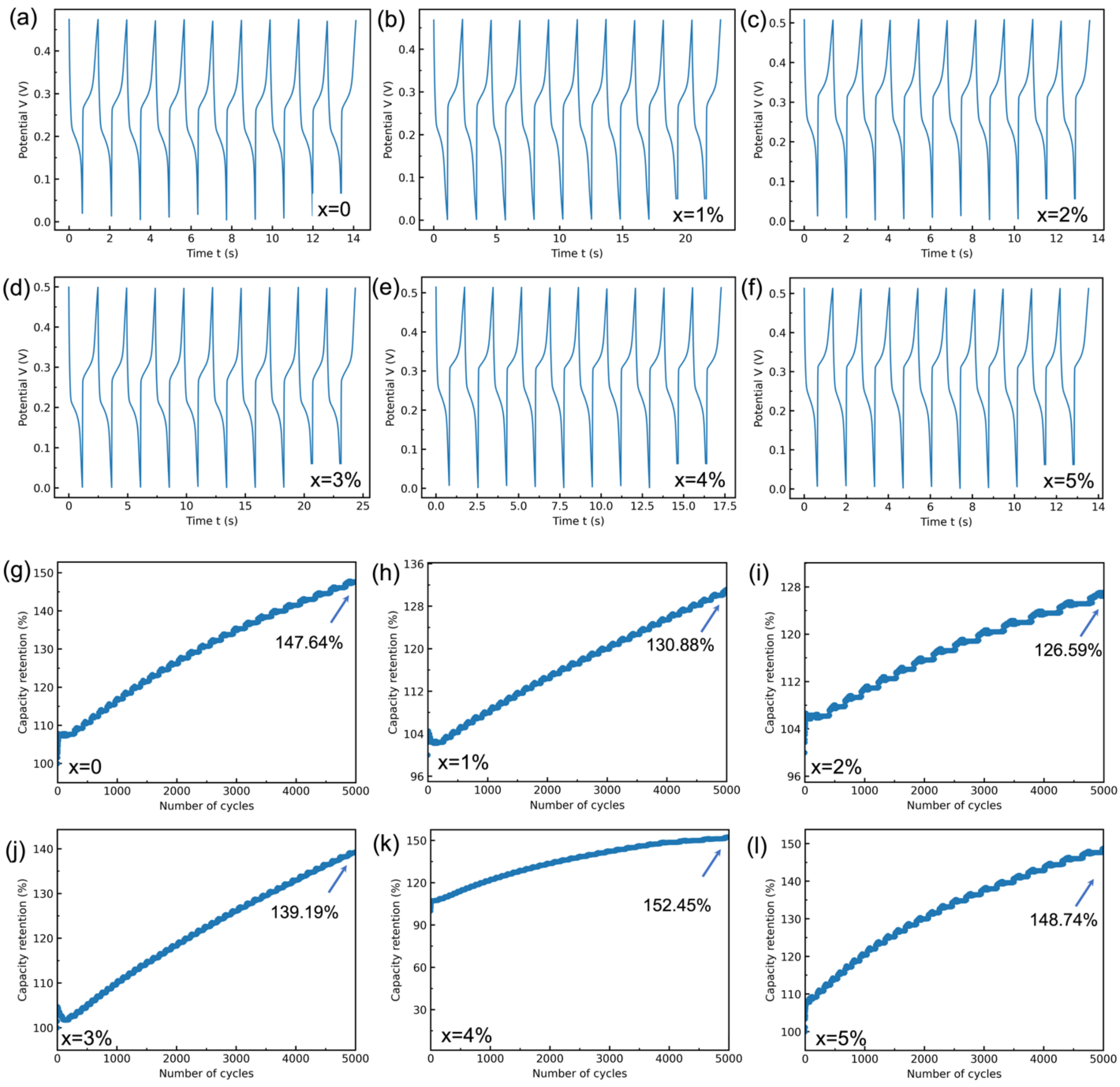

Note S3: EIS Experimental Modeling and Equivalent Circuit Considerations

1. Model Rationale

For CuO and its Ni-doped derivatives, the EIS spectra, spanning 1 mHz to 1 MHz, reveal a single major semicircle followed by a sloping low-frequency tail, characteristic of a porous electrode exhibiting both charge-transfer and diffusive processes. The optimal EC that reproduces these features was found to be:

$$Z(\omega) = R_s + j\omega L + (R_{ct} \parallel CPE_1) + CPE_2 \tag{S12}$$

In the equivalent circuit, R_s represents the uncompensated series resistance (electrolyte resistance plus current collector/contact contributions), and L accounts for the small parasitic inductance from wiring and fixtures at high frequency. The interfacial arc is described by R_{ct} in parallel with CPE_1, where CPE_1 captures the non-ideal capacitive/pseudocapacitive response caused by surface roughness, particle-size dispersion, and a distribution of relaxation times. The low-frequency tail is associated with ion-transport and diffusion polarization in the porous electrode/electrolyte-filled microstructure and is represented phenomenologically by CPE_2 (Warburg-like behavior). This mapping enables direct interpretation of the Nyquist arc diameter as an interfacial kinetic descriptor (R_{ct}) and the low-frequency slope as a transport descriptor.

2. Mathematical Formulation

The total impedance is expressed as:

$$Z(\omega) = R_s + j\omega L + \frac{1}{\frac{1}{R_{ct}} + Q_1 (j\omega)^{\alpha_1}} + \frac{1}{Q_2 (j\omega)^{\alpha_2}} \tag{S13}$$

With Q_1 (i=1, 2) being the pseudo-capacitance coefficients ($S \cdot s^{\alpha}$). α_i being the phase-angle exponents (0 < α ≤ 1). When α = 1, the CPE reduces to an ideal capacitor with $C = Q$; when α

< 1, the element expresses a dispersion of relaxation times, indicative of fractal or heterogeneous interfaces. The complex admittance of a CPE is:

$$Y_{CPE} = Q(j\omega)^{\alpha} = Q\omega^{\alpha}\left[cos\frac{\pi\alpha}{2} + jsin\frac{\pi\alpha}{2}\right] \tag{S14}$$

leading to the equivalent impedance $Z_{CPE} = 1/Y_{CPE}$Z.

3. Frequency-Domain Relationships and Derived Quantities

For a parallel combination of R_{ct} and CPE_1, the imaginary component attains a maximum at angular frequency ω_m satisfying:

$$\omega_m = \left[\frac{\tan\left(\pi(1-\alpha_1)/2\right)}{R_{ct}Q_1}\right]^{1/\alpha_1} \tag{S15}$$

The corresponding frequency is:

$$f_{max} = \frac{\omega_m}{2\pi} \tag{S16}$$

This relation allows extraction of an effective capacitance at the apex of the semicircle:

$$C_{eff} = Q_1\omega_m^{\alpha_1 - 1} \tag{S17}$$

where C_{eff} represents the dynamic capacitance associated with charge accumulation across the space-charge region and the Helmholtz layer.

4. Computational and Fitting Procedure

4.1 Data Pre-Processing

Each dataset (x = 0 to 5 %) was acquired as frequency–impedance triplets. The raw data were filtered to retain only positive, finite frequencies, followed by sorting in ascending order of frequency. Identical frequency points, often repeated due to instrument averaging, were merged by the arithmetic

mean of real and imaginary components. To balance resolution and numerical stability during optimization, logarithmic thinning was applied, dividing the frequency axis into 40–80 log-spaced bins and retaining representative midpoints. This reduced the dataset to ≈ 300 points per sample without loss of essential spectral features.

4.2 Optimization Method

Fitting was performed via nonlinear least squares using the *trust-region reflective* algorithm (scipy.optimize.least_squares) with a robust *soft-l₁* loss function to suppress outlier influence. The cost function minimized was:

$$\chi^2 = \sum_{i=1}^{N} \omega_i \left[\Re(Z'_{exp} - Z'_{mod})^2 + \Im(Z''_{exp} - Z''_{mod})^2 \right] \tag{S18}$$

where weights ω_i were defined as a product of two empirical terms, a mid-band weight emphasizing the central-frequency arc ($\omega_m = [1 + (logf_i - logf_{med})^2]^{-1}$), a magnitude normalization $\omega_m = [1 + (|Z_i|/|Z_{med}|)^2]^{-1}$.

The initial parameter vector $\theta_0 = [R_s, L, R_{ct}, Q_1, \alpha_1, Q_2, \alpha_2]$ was estimated from percentile analysis of the Nyquist plot, using low- and high-frequency limits of Z' for R_s and $R_s + R_{ct}$ respectively, and typical $\alpha \approx 0.8$ for rough surfaces.

Bounds were imposed:

$$0 \leq R_s \leq 5 \times 10^3 \Omega, \;\; 10^{-3} \leq R_{ct} \leq 2 \times 10^5 \Omega, \;\; 0.3 \leq \alpha \leq 1.0 \tag{S19}$$

Randomized restarts (Eq. S13–18) around θ_0 were employed to avoid local minima, each perturbed logarithmically for Q and R parameters and linearly for α and R_s.

Figure S13. Bode plots of Ni-doped CuO nanoparticles (x = 0–5%) showing the frequency dependence of impedance magnitude ($|Z|$) from electrochemical impedance spectroscopy (EIS). (a)–(f) correspond to doping levels of x=0 to 5%, respectively. The blue open circles represent experimental data, while the orange lines show fitted curves using the equivalent circuit model. Each plot exhibits three distinct regions: a high-frequency plateau associated with bulk resistance, a mid-frequency roll-off due to interfacial charge-transfer processes, and a low-frequency rise reflecting ionic diffusion and capacitive behavior. The progressive left-to-right shift of the mid-frequency transition indicates improved charge transport kinetics with increasing Ni content, with an optimal response observed at x = 3%.

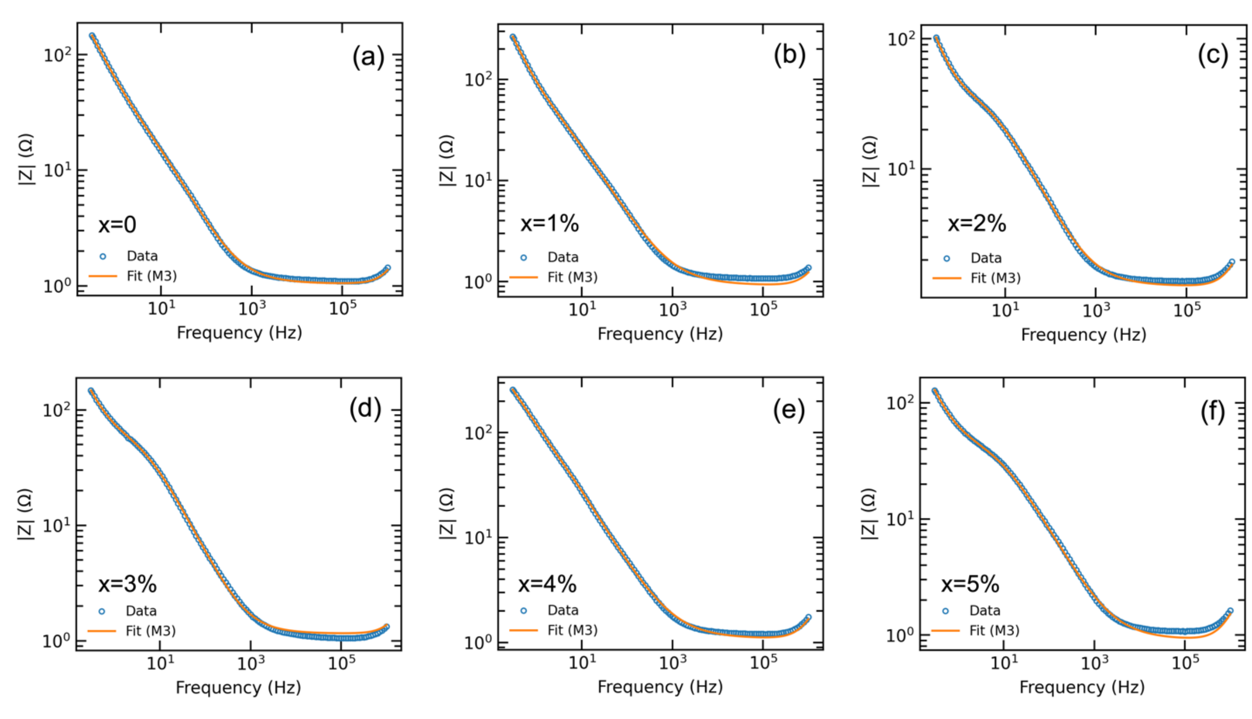

Figure S14. Phase angle (Bode phase) plots for Ni-doped CuO nanoparticles (x = 0–5%) as a function of frequency, with experimental and fitted EIS data. (a)–(f) correspond to Ni doping levels of x=0 to 5%, respectively. Blue open circles denote the measured phase response, while orange lines represent fits obtained from the equivalent circuit model. Each plot displays two primary inflection zones corresponding to distinct electrochemical processes: interfacial charge transfer and ionic accumulation. The upward shift and narrowing of the phase minimum with increasing Ni content up to x = 3% indicate enhanced dielectric homogeneity and faster relaxation dynamics. At higher doping (x ≥ 4%), a broadening of the response is observed, consistent with increased disorder or defect clustering.

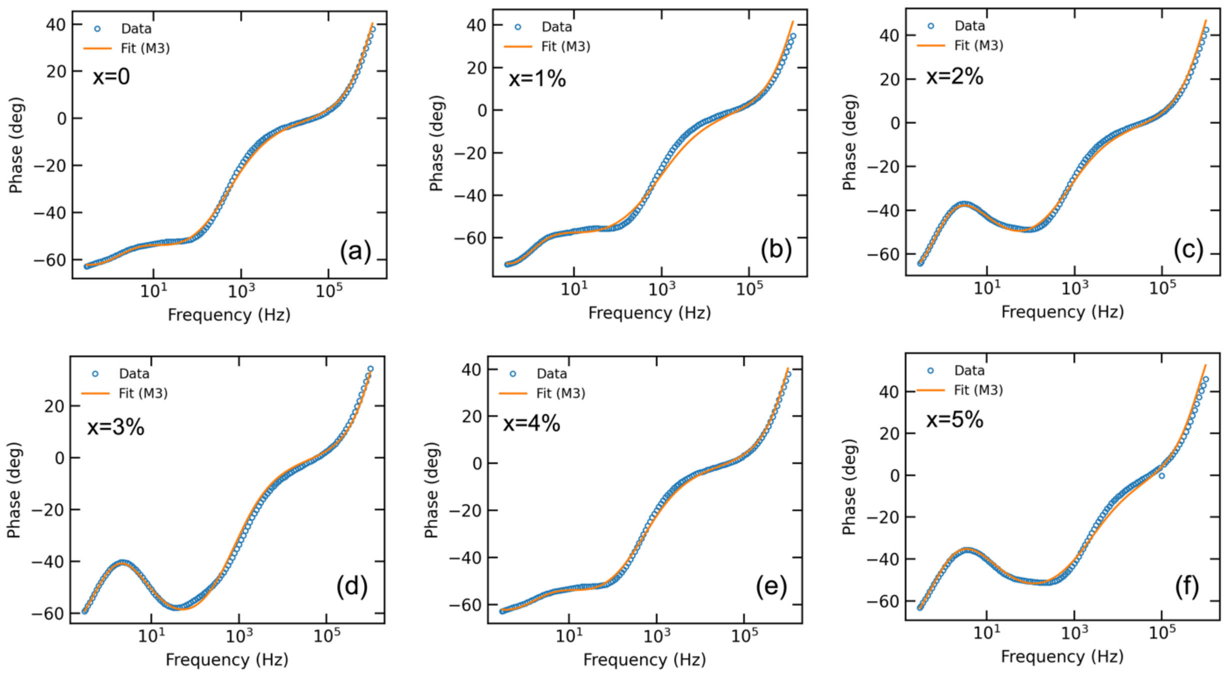

Figure S15. Residual plots of the EIS fitting for Ni-doped CuO nanoparticles (x = 0–5%), showing deviations between experimental and fitted impedance data. (a)–(f) display the residuals ($\Delta Z'$ and $\Delta Z''$) as a function of frequency for doping levels of x=0 to 5%, respectively. Blue open circles represent residuals in the real component (Z'), while orange open circles correspond to the imaginary component (Z''). The small magnitude and random distribution of residuals across most frequencies confirm the robustness and accuracy of the nonlinear least-squares fitting using the equivalent circuit model. Notably, slight deviations at low frequencies (particularly at x= 1% and 4%) may reflect limitations in capturing deep diffusive or inductive features. The residuals remain within $\pm 1\,\Omega$ for most samples, validating the model's physical relevance and numerical consistency.

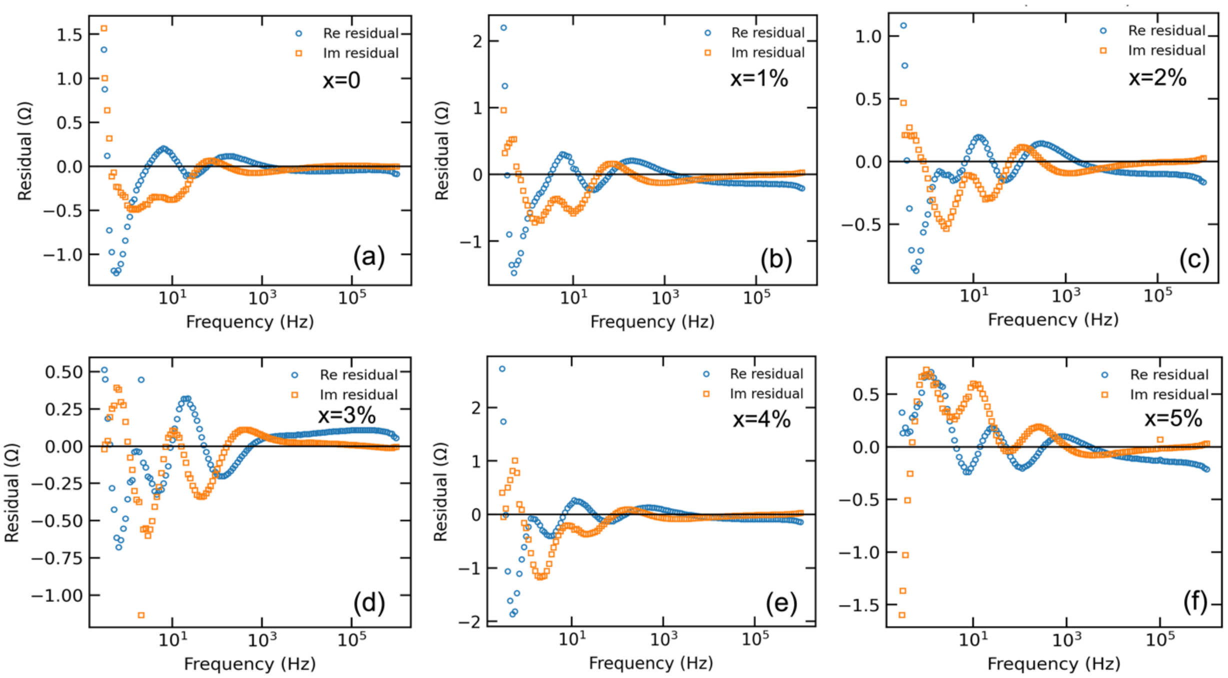

Table S1. Rietveld refinement parameters for undoped CuO and 5% Ni-doped CuO nanoparticles. Values in parentheses denote the estimated standard uncertainties (1σ) from the refinement output. The results indicate a slight lattice expansion and an increase in structural disorder upon Ni doping. Additional quantitative phase analysis (Hill–Howard method) indicates a minor NiO secondary phase.

x(%)	a (Å)	b (Å)	c (Å)	$\alpha(°)$	$\beta(°)$	$\gamma(°)$	Volume (Å^3)	$R_{wp}(\%)$	$R_p(\%)$	χ^2
0	4.6874(4)	3.4268(3)	5.1280(5)	90	99.319(4)	90	81.289(12)	14.3	11.9	1.480
5	4.6890(2)	3.4328(1)	5.1378(2)	90	99.342(3)	90	81.603(5)	10.6	9.29	1.530

Table S2. ML-extracted parameters from SEM images.

Metric	CuO nanoparticles	5% Ni–CuO nanoparticles
Scale (nm px^{-1})	0.735	0.735
Particles analyzed, n	226	221
Mean ECD d_{eq} (nm)	27.76	28.88
Median ECD, D50 (nm)	25.41	26.68
Std. dev. (nm)	14.64	14.58
D10 (nm)	10.85	10.79
D90 (nm)	48.38	50.26
Lognormal μ (ln nm)	3.1801	3.2214
Lognormal σ	0.5522	0.5541
Geometric mean (nm)	24.05	25.06
Geometric std factor (×)	1.74	1.74
KS D, p-value	≈ 0.055, ≈ 0.49	≈ 0.062, ≈ 0.35
Mann–Whitney (a vs b)	—	U = 23674.5, p = 0.342, r_{rb}=0.052

Table S3. Elemental composition of CuO and Ni-doped CuO nanoparticles by EDX.

Sample	Cu (at%)	O (at%)	Ni (at%)
CuO (undoped)	51.3	48.7	–
CuO:Ni (5%)	47.9	47.2	4.9

Note: Values are semi-quantitative and normalized to 100 at%. Oxygen quantification may carry higher uncertainty due to detector sensitivity and surface effects. Ni values correspond to nominal doping concentrations and confirm successful substitution of Cu by Ni in the lattice.

Table S4. Comparison of experimental and theoretical Raman-active phonon Modes of CuO.

Mode Symmetry	Assignment	Experimental (This Work) [cm^{-1}]	DFT (Ref. S1) [cm^{-1}]	Remarks
A_g	Cu–O bending	297	292	Sensitive to lattice distortion
$B_g(1)$	Cu–O bond deformation	346	341	Shifts with Ni doping
$B_g(2)$	Cu–O stretching along [101]	629	624	Most intense mode
–	Two-phonon overtone/combination	~1050	Not reported (2nd order)	Observed in doped samples

Note: DFT data from Bielecki et al.[S1] using GGA+U approximation.

Table S5. Combined fitting parameters from GCD analysis for Ni-doped CuO nanoparticles (x=0 to 5%) over 1–5 $A \cdot g^{-1}$. For each composition and current density, the table reports the selected discharge fitting window (start/end time and Δt), voltage bounds (V_{start}, V_{end}, ΔV), linear fit coefficients (slope and intercept), the absolute discharge rate $\alpha = | dV/dt |$, the specific capacitance $C_{sp} = J/(\alpha)$ (with J in $A \cdot g^{-1}$), and fit quality R^2. Windowing rules: x=0 to 5% use a R^2-max sliding window constrained to the central portion of the discharge (IR drop and deep tail excluded; minimum window length ≥ 30% of the discharge). Time is referenced to the discharge apex for each cycle. Reported R^2 values quantify linearity within the selected window; uncertainties are dominated by window selection and baseline drift and are discussed in the main text.

x (%)	Current density J ($A \cdot g^{-1}$)	t_{start} (s)	t_{end} (s)	Δt (s)	V_{start} (V)	V_{end} (V)	ΔV (V)	$V_{intercep}$ (V)	α ($V \cdot s^{-1}$)	R^2	C_{sp} ($F \cdot g^{-1}$)
0	1	0.9604	33.0504	32.09	0.4274	0.0487	0.3787	0.361	0.0082	0.9323	121.9337
0	2	0.4224	16.5234	16.101	0.4275	0.0475	0.38	0.358	0.0169	0.9337	118.5987
0	3	0.2375	10.6595	10.422	0.4275	0.0475	0.38	0.3555	0.0264	0.9369	113.6419
0	4	0.1494	7.7264	7.577	0.4275	0.0475	0.38	0.3536	0.0369	0.9387	108.4283
0	5	0.1015	5.9415	5.84	0.4273	0.0474	0.3799	0.3516	0.0481	0.9378	103.902
1	1	0.3704	21.1304	20.76	0.4229	0.0478	0.3751	0.3412	0.0123	0.9331	81.4713
1	2	0.1514	9.6114	9.46	0.4229	0.047	0.3758	0.3391	0.0271	0.9337	73.8648
1	3	0.0805	5.8915	5.811	0.4229	0.0471	0.3759	0.3368	0.0448	0.9394	66.9993
1	4	0.0505	4.1075	4.057	0.4227	0.047	0.3757	0.3359	0.0656	0.9441	60.9538
1	5	0.0345	3.0705	3.036	0.4224	0.047	0.3754	0.3357	0.0891	0.9462	56.1116
2	1	1.4004	23.7304	22.33	0.4588	0.0516	0.4072	0.4576	0.0129	0.9411	77.4653
2	2	0.5664	11.9114	11.345	0.459	0.0509	0.4081	0.4504	0.0266	0.9571	75.1152
2	3	0.3004	7.6824	7.382	0.459	0.051	0.408	0.4465	0.0428	0.9523	70.0368
2	4	0.1804	5.4114	5.231	0.4589	0.0509	0.408	0.4397	0.0594	0.9473	67.3497
2	5	0.1184	4.0884	3.97	0.4589	0.051	0.4079	0.4336	0.0765	0.9444	65.3514
3	1	1.2114	21.4014	20.19	0.4589	0.0517	0.4072	0.4615	0.0145	0.9475	68.8045
3	2	0.4864	10.7334	10.247	0.459	0.0509	0.4081	0.4573	0.0313	0.958	63.8662
3	3	0.2474	6.5904	6.343	0.459	0.051	0.408	0.45	0.0501	0.9496	59.8651
3	4	0.1434	4.5754	4.432	0.4591	0.0511	0.4079	0.4423	0.0696	0.9438	57.499
3	5	0.0914	3.4484	3.357	0.4588	0.051	0.4078	0.4358	0.0898	0.9393	55.6891
4	1	0.6044	12.3444	11.74	0.4632	0.0528	0.4104	0.4679	0.0264	0.9484	37.8704
4	2	0.2094	5.6934	5.484	0.4634	0.0514	0.412	0.4568	0.0576	0.9541	34.7307

4	3	0.1004	3.4254	3.325	0.4634	0.0516	0.4118	0.449	0.0954	0.9477	31.4482
4	4	0.0574	2.3284	2.271	0.4635	0.0517	0.4118	0.441	0.1357	0.9405	29.4695
4	5	0.0374	1.7194	1.682	0.463	0.0514	0.4116	0.4336	0.1778	0.9344	28.1162
5	1	1.5574	29.4274	27.87	0.4634	0.0518	0.4116	0.456	0.0103	0.9472	96.662
5	2	0.5984	14.3874	13.789	0.4635	0.0516	0.4119	0.4493	0.0222	0.9502	89.9017
5	3	0.3024	8.9174	8.615	0.4635	0.0515	0.412	0.4421	0.0357	0.9419	84.1325
5	4	0.1764	6.2034	6.027	0.4634	0.0516	0.4119	0.4343	0.0498	0.9355	80.3941
5	5	0.1114	4.6534	4.542	0.4634	0.0516	0.4118	0.4274	0.0647	0.93	77.2428

Table S6. Summary of fitting parameters for Ni-doped CuO nanoparticles (x =0 to 5%) over *various current density* $J = 1 - 5\, A \cdot g^{-1}$. Discharge window bounds (times and voltages), linear-fit coefficients, J , Δt, and V_{avg}, were used to derive the window-based energy and power densities.

x (%)	Current density J (A/g)	Δt (s)	ΔV (V)	V_{avg} (V)	E_{sp} $(Wh \cdot kg^{-1})$	P_{sp} $(kW \cdot kg^{-1})$
0	1	32.09	0.3787	0.23805	2.122	0.0661
0	2	16.101	0.38	0.2375	2.1244	0.1319
0	3	10.422	0.38	0.2375	2.0627	0.1979
0	4	7.577	0.38	0.2375	1.9995	0.2639
0	5	5.84	0.3799	0.23735	1.9252	0.3297
1	1	20.76	0.3751	0.23535	1.3572	0.0654
1	2	9.46	0.3758	0.23495	1.2348	0.1305
1	3	5.811	0.3759	0.235	1.138	0.1958
1	4	4.057	0.3757	0.23485	1.0587	0.261
1	5	3.036	0.3754	0.2347	0.9897	0.326
2	1	22.33	0.4072	0.2552	1.5829	0.0709
2	2	11.345	0.4081	0.25495	1.6069	0.1416
2	3	7.382	0.408	0.255	1.5687	0.2125
2	4	5.231	0.408	0.2549	1.4815	0.2832
2	5	3.97	0.4079	0.25495	1.4058	0.3541
3	1	20.19	0.4072	0.2553	1.4318	0.0709
3	2	10.247	0.4081	0.25495	1.4514	0.1416
3	3	6.343	0.408	0.255	1.3479	0.2125
3	4	4.432	0.4079	0.2551	1.2562	0.2834
3	5	3.357	0.4078	0.2549	1.1885	0.354
4	1	11.74	0.4104	0.258	0.8414	0.0717
4	2	5.484	0.412	0.2574	0.7842	0.143
4	3	3.325	0.4118	0.2575	0.7135	0.2146
4	4	2.271	0.4118	0.2576	0.65	0.2862
4	5	1.682	0.4116	0.2572	0.6008	0.3572
5	1	27.87	0.4116	0.2576	1.9943	0.0716
5	2	13.789	0.4119	0.25755	1.973	0.1431
5	3	8.615	0.412	0.2575	1.8486	0.2146
5	4	6.027	0.4119	0.2575	1.7244	0.2861
5	5	4.542	0.4118	0.2575	1.6244	0.3576

Table S7. Benchmark comparison of CuO and CuO-based (including Ni-containing) supercapacitor systems reported in aqueous electrolytes. The table summarizes the electrode/device configuration, electrolyte, operating voltage window, and the reported gravimetric energy and power densities together with cycling stability. Values are reproduced as reported in the cited references (device-level unless explicitly noted as three-electrode/electrode-level), and differences in cell configuration and voltage window should be considered when comparing absolute energy densities. The present Ni–CuO electrode is included for direct reference under the same aqueous test conditions.

System	Configuration	Electrolyte	Voltage (V)	E_{sp} $(Wh \cdot kg^{-1})$	P_{sp} $(W \cdot kg^{-1})$	Cycling stability	Ref.
$Cu_{1-x}Ni_xO$ (x = 2% $J = 2\ A \cdot g^{-1}$)	3-electrodes	3 M KOH	0–0.55	1.61	141.6	126.59% after 5000 cycles	This work
Cu_xO/Cu foil	Asymmetric device	1 M KOH	0.6–1.6	24.20	650	94.7% after 12000 cycles	S2
CuO@Cu_4O_3/rGO/PANI	Symmetric device	1 M KOH	−1.0–0.5	23.95	374	93% after 5000 cycles	S3
CuO@MnO_2//MEGO	Asymmetric device	1 M Na_2SO_4	1.8	22.1	85,600	101.5% after 10000 cycles	S4
CuO/rGO@NF	Symmetric device	6 M KOH	not stated	38.35	187.5	70.7% after 2000 cycles	S5

CuO/NiO NCs	Electrode/device (reported)	1 M NaOH	—	4.24	10,670	90% after 2000 cycles	S6
CuO@ZnCo–OH//LSDC	Asymmetric device	aqueous (reported)	1.5	22.10	434	91.7% after 4000 cycles	S7
CuO/CFF	Device (reported)	aqueous (reported)	—	10.05	1,798.5	—	S8

References

S1. J. Bielecki, et al., Phonon properties of CuO: density functional theory and Raman spectroscopy study, Physical Review B **2014**, 89, 155117. DOI:10.1103/PhysRevB.89.155117.

S2. Li, Z.; Lin, J.; He, X.; Xin, Y.; Liang, P.; Zhang, C. Cu_xO-Modified Nanoporous Cu Foil as a Self-Supporting Electrode for Supercapacitor and Oxygen Evolution Reaction. Nanomaterials **2022**, 12, 2121. DOI: 10.3390/nano12122121.

S3. Allah, A. E.; *et al.* Chemical synthesis and super capacitance performance of novel CuO@Cu_4O_3/rGO/PANI nanocomposite electrode. RSC Adv. **2024**, 14, 13628-13639. DOI: 10.1039/D4RA00065J.

S4. Huang, M.; *et al.* Merging of Kirkendall Growth and Ostwald Ripening: CuO@MnO_2 Core–Shell Architectures for Asymmetric Supercapacitors. Sci. Rep. **2104**, 4, 4518. DOI: 10.1038/srep04518.

S5. Zhai, M.; *et al.* CuO nanorods grown vertically on graphene nanosheets as a battery-type material for high-performance supercapacitor electrodes. RSC Adv. **2020**, 10, 36554. DOI: 10.1039/D0RA06758J.

S6. Arulkumar, E.; Shanthosh Shree, S.; Thanikaikarasan, S. Structure, morphology, composition, optical properties of CuO/NiO nanocomposite for electrochemical energy storage devices. Results in Chemistry **2023**, 6, 101087. DOI: 10.1016/j.rechem.2023.101087.

S7. Liu, G.; Song, X.-Z.; Zhang, S.; Chen, X.; Liu, S.; Meng, Y.; Tan, Z. Hierarchical CuO@ZnCo–OH core-shell heterostructure on copper foam as three-dimensional binder-free electrodes for high performance asymmetric supercapacitors. J. Power Sources **2020** 465, 228239 (2020). DOI: 10.1016/j.jpowsour.2020.228239.

S8. Xu, W.; Dai, S.; Liu, G.; Xi, Y.; Hu, C.; Wang, X. CuO Nanoflowers Growing on Carbon Fiber Fabric for Flexible High-Performance Supercapacitors. Electrochim. Acta **2016**, 203,1. DOI: 10.1016/j.electacta.2016.03.170.
